# Molecular epidemiology of HIV-1 in Hungary: an evolving contact zone of colliding virus subtypes

**DOI:** 10.3389/fmicb.2025.1732254

**Published:** 2025-12-16

**Authors:** Levente Zsichla, Lilla Adravecz, Dalma Müller, Philippe Lemey, Áron Lakatos, Eszter Ari, Katharina Kusejko, Roger Kouyos, János Szlávik, Botond Lakatos, Éva Áy, Viktor Müller

**Affiliations:** 1 Institute of Biology, ELTE Eötvös Loránd University, Budapest, Hungary; 2 National Laboratory for Health Security, ELTE Eötvös Loránd University, Budapest, Hungary; 3 National Reference Laboratory for Retroviruses, Department of Virology, National Center for Public Health and Pharmacy, Budapest, Hungary; 4 Department of Bioinformatics, Semmelweis University, Budapest, Hungary; 5 Department of Microbiology, Immunology and Transplantation, Rega Institute, KU Leuven, Leuven, Belgium; 6 Department of Infectology, Central Hospital of Southern Pest, National Institute of Hematology and Infectious Diseases, National Center of HIV, Budapest, Hungary; 7 Department of Genetics, ELTE Eötvös Loránd University, Budapest, Hungary; 8 Synthetic and Systems Biology Unit, Institute of Biochemistry, HUN-REN Biological Research Centre, Szeged, Hungary; 9 HUN-REN Office for Supported Research Groups, Budapest, Hungary; 10 Department of Infectious Diseases and Hospital Epidemiology, University Hospital Zurich, University of Zurich, Zurich, Switzerland; 11 Institute of Medical Virology, University of Zurich, Zurich, Switzerland; 12 Department of Internal Medicine and Hematology, Departmental Group of Infectious Diseases, Semmelweis University, Budapest, Hungary

**Keywords:** molecular epidemiology, HIV, phylogenetics, phylogeography, transmission cluster analysis

## Abstract

**Introduction:**

Although antiretroviral therapy can suppress the transmission of HIV-1, the pandemic persists and continues to evolve. Monitoring virus transmission patterns and evolving variants is therefore essential for improving prevention strategies. To address this need, we present the first comprehensive molecular epidemiological analysis of the HIV-1 epidemic in Hungary—a country in the contact zone of major HIV-1 subtypes A, B, and F.

**Methods:**

We analyzed partial *pol* sequences obtained from 1,120 Hungarian patients in the context of routine drug resistance genotyping between 2008 and 2024, along with 2,202 international background sequences selected based on sequence similarity. We performed subtyping, drug resistance testing, maximum likelihood and Bayesian phylogenetic inference, distance-based and phylogenetic clustering, and Bayesian phylogeographic analyses to identify domestic clusters and cross-border transmission.

**Results:**

Most sequences (814/1,120) belonged to subtype B; however, the frequency of non-B subtypes (mainly A, F, and several recombinant forms) has increased steadily since 2014, reaching 41.3% in 2024. Phylogenetic analyses identified 136 domestic clusters and a large transmitted drug resistance clade persisting over two decades. Remarkably, one in six recent diagnoses mapped to a single cluster, and approximately a third of all new diagnoses to the five most active clusters. Individuals in larger clusters were more often young, men who have sex with men (MSM), and had higher CD4+ counts. While the most affected risk group is still MSM, suspected heterosexual cases have increased recently, with a clear separation between sub-epidemics. Incorporation of international sequences revealed 149 mixed clusters, 56 mixed monophyletic pairs, and at least 122 independent introductions, linking the Hungarian epidemic predominantly to other European countries (Germany, UK, Poland, Spain, Italy, and Croatia for subtype B; Russia and Poland for subtype A; Romania for subtype F, Italy for CRF18_cpx), and to more distant sources for CRF01_AE (Thailand, China), CRF02_AG (Spain, Cameroon) and CRF56_cpx (Turkey).

**Discussion:**

In summary, recent HIV infections in Hungary stem mainly from domestic transmission among MSM, with a few highly active transmission clusters underscoring the need for targeted interventions. The epidemic is most strongly linked to Western and Central Europe, with increasing introductions and spread of non-B subtypes from Eastern Europe and beyond.

## Graphical Abstract

**Figure:**
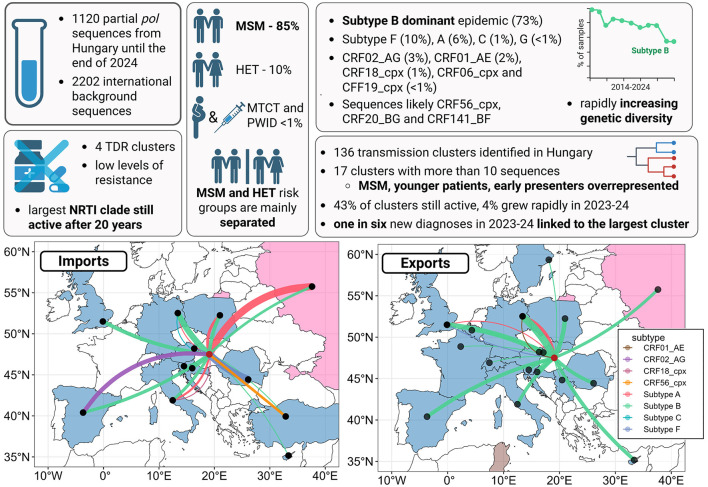
Created in BioRender. Zsichla, L. (2025) https://BioRender.com/za6ltyr.

## 1 Introduction

Human Immunodeficiency Virus type 1 (HIV-1) is characterized by high genetic diversity, primarily due to its error-prone reverse transcriptase and the absence of proofreading mechanisms (Roberts et al., 1988). The strain of HIV-1 responsible for the global pandemic, known as group M, has diversified into 10 recognized subtypes (A–D, F–H, and J–L) (Nair et al., 2024), which, due to distinct founder effects and subsequent local transmission (Ferdinandy et al., 2015), have become predominant in specific geographical regions. In addition, recombination between HIV-1 strains further contributes to genetic variability, leading to the emergence of 167 known circulating recombinant forms (CRFs) as of November 2025, which demonstrate evidence of sustained transmission, in contrast to unique recombinant forms (URFs) (LANL Sequence Database, 2025). This extensive genetic diversity facilitates the tracking of transmission dynamics within and between population groups (Beloukas et al., 2016; Nduva et al., 2021) and the identification of case clusters at both national and international levels (Hassan et al., 2017; Grabowski et al., 2018).

Hungary, a country with a population of 9.5 million, has one of the lowest rates of HIV diagnoses in the WHO European Region, with 2.1–2.7 registered cases per 100,000 inhabitants annually since 2014 and a cumulative total of 5,123 HIV and 1,285 AIDS diagnoses from 1986 to the end of 2024 (European Centre for Disease Prevention Control, 2024). Approximately 9 out of 10 diagnosed individuals were male, and the most prevalent risk group was men who have sex with men (MSM, 77.9% of cases). Other documented transmission routes—heterosexual contact (HET, 18.4%), people who inject drugs (PWID, 1.0%), and mother-to-child transmission (MTCT, 0.6%)—represent less prevalent risk categories (National Center for Public Health Pharmacy, 2025). Similar to other MSM-dominant epidemics in Europe, the HIV-1 epidemic in Hungary has historically been dominated by subtype B; however, several non-B subtypes began to increase in prevalence in the mid-2010s (Áy et al., 2020).

Hungary is situated at the intersection of several epidemiologically distinct regions, which makes it an ideal sentinel country to detect geographical patterns of international HIV spread or the expansion of (potentially) more transmissible subtypes. Neighboring countries to Hungary's north, west, and south—Austria (Leierer et al., 2024), Slovakia (Habekova et al., 2010), Slovenia (Lunar et al., 2015, 2018), Croatia (Oroz et al., 2019; Planinić et al., 2023), and Serbia (Jovanović et al., 2019)—exhibit low-prevalence HIV epidemics that are dominated by subtype B and concentrated in the MSM risk group, similar to the epidemic in Hungary (European Centre for Disease Prevention Control, 2024). In contrast, Ukraine, located at Hungary's northeastern border and at the edge of the subtype A-prevalent region of Eastern Europe (particularly affected by the A6 lineage), exhibits a higher overall HIV prevalence, with a larger proportion of cases among PWID and HET individuals (Vasylyeva et al., 2018, 2019). The ongoing war has resulted in large-scale displacement of Ukrainian citizens since 2022, mainly toward Russia, Poland, Romania, the Republic of Moldova, Slovakia, and Hungary (Lloyd and Sirkeci, 2022), potentially facilitating cross-border transmission of the A6 sub-subtype (Serwin et al., 2023, 2024; Weibull Wärnberg et al., 2023). Finally, to the east, Romania has another distinct epidemiological profile, uniquely dominated by subtype F (Preda and Manolescu, 2022). The country also has a Hungarian ethnic minority population exceeding 1 million individuals, and accounts for the largest number of non-citizen residents in Hungary (Kincses, 2015).

This unique epidemiological context implies that the genomic surveillance of HIV-1 in Hungary has relevance at the regional and international levels and can provide early warning signals for the cross-regional transmission of expanding HIV-1 lineages. Leveraging data from the national routine drug resistance monitoring system, we present the first large-scale molecular epidemiological analysis of the HIV-1 epidemic in Hungary until the end of 2024, seeking to identify transmission clusters, epidemiological links to and from other countries, and analyze the shifting dominance of HIV-1 subtypes and the compartmentalization of risk groups.

## 2 Materials and methods

### 2.1 Study population and sequencing

In this nationwide molecular epidemiological study, we analyzed sequences of 1,146 peripheral blood samples collected from 1,120 individuals (diagnosed between 1993 and 2024) living with HIV-1 between 2008 and 2024, as part of routine HIV-1 genotyping. In our analyses, we retained the first sample per patient, resulting in a final set of 1,120 analyzed sequences.

Serving as a primary HIV Center in Hungary, the National Institute of Hematology and Infectious Diseases in the Central Hospital of Southern Pest provided the majority of blood samples used to obtain the HIV-1 sequences included in this study. To enhance the comprehensiveness and geographic coverage of our dataset, additional samples were contributed from a clinic at Semmelweis University and two smaller HIV treatment centers in Pécs and Debrecen, both established in or after 2014. Most of the samples were obtained from newly diagnosed, antiretroviral therapy (ART)-naïve patients living with HIV-1; however, ART-experienced individuals exhibiting detectable HIV-1 viral load and patients with known pre-exposure prophylaxis (PrEP) usage were also included in this study.

Sample processing and Sanger sequencing of the viral protease (PR), partial reverse transcriptase (RT), and integrase (INT) genomic regions were performed by the National Reference Laboratory for Retroviruses at the National Center for Public Health and Pharmacy, Budapest, Hungary, in accordance with previously described protocols (Mezei et al., 2011; Áy et al., 2020, 2021).

### 2.2 Subtyping and drug resistance analyses

For HIV-1 subtype determination, the PR, RT and INT coding sequences were analyzed using the Stanford HIVDB subtyping program (Rhee et al., 2003), the REGA (de Oliveira et al., 2005) and the COMET tools (Struck et al., 2014). The final subtype assignment was determined by expert opinion based on the results of the three tools. Sequences that could not be unambiguously classified were labeled as “non-typable”. The presence and clinical relevance of drug resistance mutations (DRMs) were assessed by the Stanford HIV Drug Resistance Database algorithm (Liu and Shafer, 2006); mutations classified as surveillance drug resistance mutations (SDRMs) that formed clusters of three or more sequences on the maximum likelihood phylogenetic tree of sequences sampled in Hungary were defined as transmitted drug resistance (TDR) clades in our analysis.

### 2.3 Maximum likelihood phylogenetic analysis

The genomic region covering partial PR and RT (PRRT) was amplified as a single fragment in most samples, while in a subset of samples, it was generated as two contigs. These contigs were then concatenated by inserting indeterminate “N” nucleotides at the missing positions (primarily between codons RT13-32), based on alignment with the HXB2/K03455 reference sequence. Additionally, the full INT region of the genome was amplified for most samples.

Three maximum likelihood phylogenetic trees were built from the sequences sampled in Hungary. The first tree, used to characterize domestic transmission in Hungary, included all sequences sampled in Hungary and a reference dataset encompassing HIV-1 group M subtypes (A, B, C, D, F, and G) and circulating recombinant forms (CRFs) possibly present in Hungary based on initial subtyping results (Los Alamos Sequence Database, Recombination Identification Program Alignment 2023, https://www.hiv.lanl.gov/content/sequence/NEWALIGN/help.html#RIP, accessed on 13 January 2025). The second tree—to identify transmission clusters specific to the country and possible epidemiological links to other countries—additionally to the sequences from Hungary and the reference dataset, included an additional set of background control sequences. This set was constructed by pooling the five most similar NCBI BLASTN (megablast) (Zhang et al., 2000) search results against all HIV sequences in NCBI GenBank for each of the partial *pol* sequences obtained in Hungary. All sequences submitted previously to GenBank from Hungary were excluded, as these are already reported in (Mezei et al., 2011; Áy et al., 2020). GenBank was queried separately for PRRT, INT, and concatenated PRRT and INT sequences (concatenated in the same way as described previously for PR and partial RT sequences). The resulting three sequence sets were combined and deduplicated before further analysis. The third phylogenetic tree included all sequences sampled in Hungary classified as “non-typable” based on subtyping results, and the whole Recombination Identification Program Alignment to determine the subtype of ambiguously subtyped sequences.

Multiple sequence alignments were performed separately for PRRT and INT sequences using MAFFT v7.490 (Katoh and Standley, 2013) [with five cycles of iterative refinement (- -maxiterate 5)]. Nucleotide positions associated with the most common drug resistance mutations, based on the Stanford drug resistance mutation lists (Liu and Shafer, 2006), were excluded to avoid the confounding effect of ART-driven convergent evolution. Additionally, nucleotide positions at both ends of the alignments that were either absent or indeterminate in at least 50% of the sequences from Hungary or in the combined dataset were trimmed. The PRRT and INT alignments were manually inspected and corrected using Aliview v1.28 (Larsson, 2014). After this step, the alignments were concatenated into a unified PRRT-INT dataset, combining the two genomic fragments. The total alignment length for the dataset including only sequences from Hungary was 2,084 base pairs (918 bp for the PRRT and 1,166 bp for the INT region). Sequences from Hungary covered HXB2 positions 2,268-3,308 (codons PR6-RT253) and 4,044-5,242 (codons RNAse59-Vif63, including the full integrase region INT1-288). With the inclusion of international background sequences, the total alignment length increased to 2,137 bp (945 bp for the PRRT and 1,192 bp for the INT region).

Maximum likelihood phylogenetic trees were constructed using RAxML-NG v1.2.2 (Kozlov et al., 2019) under the GTR+Γ model with partitioned analysis. Branch support values were calculated using the automated bootstrap method (- -autoMRE) in RAxML-NG, with a maximum of 500 replicates.

The steps of the data pipeline were automated using scripts implemented in GNU bash v5.1.16, Python v3.10.11 (Van Rossum and Drake, 2009), and R v4.3.1 (R Core Team, 2023).

### 2.4 Transmission cluster analyses

In our main analysis, potential transmission clusters were identified using ClusterPicker v1.2.5 (Ragonnet-Cronin et al., 2013) applied on phylogenetic trees and HIV-TRACE v0.8.0 (Kosakovsky Pond et al., 2018) on multiple sequence alignments; we then considered the union of clusters identified by both methods. ClusterPicker was applied with a maximum genetic distance threshold of 0.045 nucleotide substitutions per site within clusters and a bootstrap support threshold of 80 (Novitsky et al., 2020). HIV-TRACE was set to use a maximum pairwise distance threshold of 0.01 substitutions per site (Wertheim et al., 2017; Labarile et al., 2023) and the preprocessed sequence of the HXB2 reference genome (GenBank accession number K03455.1). Sequence sets with at least two entries were classified as transmission clusters in the Hungarian-only tree, while in the tree including international sequences, clusters were defined as those with at least two sequences, including at least one from Hungary. To assess the impact of different genetic distance thresholds and branch support values on the number and size of clusters both for individual tools and for the combined assignments, we conducted sensitivity analyses comparing results with distance thresholds of 0.005, 0.0075, 0.01, 0.0125, and 0.015 for HIV-TRACE, and 0.015, 0.03, 0.045, and 0.055 for ClusterPicker. For ClusterPicker, we tested branch support thresholds of 0, 50, 80, and 95. We estimated the growth of identified transmission clusters in the Hungarian-only tree based on the distribution of diagnosis dates.

### 2.5 Phylogenetic cherry analysis

We extracted all monophyletic pairs—henceforth referred to as “cherries”—from the phylogenetic tree including international background sequences, using the R package *ape* v5.8 (Paradis and Schliep, 2019), applying a maximum phylogenetic distance of 0.045 substitutions per site (Kusejko et al., 2022). Cherries were classified into two categories: domestic cherries, where both sequences were obtained in Hungary; and mixed cherries, where one sequence originated in Hungary and the other abroad. Additionally, we calculated the assortativity factor (AF) for risk groups, defined as the ratio of expected (random pairings of tips) to observed pairings among domestic cherries. We checked the sensitivity of assortativity analysis results by repeating the cherry extraction using maximum phylogenetic distances of 0.015, 0.03, and 0.055 substitutions per site. For further details on the assortativity analysis, see (Kusejko et al., 2022).

### 2.6 Bayesian phylogenetic and phylogeographic analyses

Time-calibrated trees were built using Bayesian phylogenetic analysis in BEAST v10.5.0 (Baele et al., 2025). We used the concatenated Hungarian and international background PRRT-INT sequences described in the *Maximum likelihood phylogenetic analysis* section, supplemented with country of origin and sampling date data derived from NCBI GenBank for the background sequence set. Subtypes were analyzed separately, including only those with at least 10 sequences from Hungary (subtypes A, B, C, F, CRF01_AE, CRF02_AG, CRF18_cpx, and CRF56_cpx).

The Bayesian analysis employed the GTR+Γ nucleotide substitution model with codon partitions, combined with an uncorrelated lognormal relaxed molecular clock model and a Bayesian Skygrid coalescent tree prior. Ancestral state reconstruction used an asymmetric substitution model. We implemented a Bayesian stochastic search variable selection (BSSVS) analysis to investigate migration flows, simultaneously obtaining Markov jumps indicating import events to, or export from Hungary. Priors for the mean clock rate parameters were estimated based on the dataset of Bletsa et al. (2019).

Markov chain Monte Carlo analyses were run for 10^7^ to 10^8^ generations, depending on dataset size. Convergence was assessed using Tracer v1.7.2 (Rambaut et al., 2018); the first 10% of samples were discarded as burn-in, and the effective sample size threshold for all parameters was set to 200.

The maximum clade credibility (MCC) tree was generated using TreeAnnotator v1.10.4 (Helfrich et al., 2018) with node heights based on common ancestor heights. The timing of introduction events into Hungary was estimated using the median node heights, the 95% highest posterior density intervals of the most recent common ancestor (MRCA) of Hungarian clades and the MRCA of these clades with their closest international background sequence(s).

### 2.7 Statistical analyses

To compare trait frequencies between samples with B and non-B subtype sequences we used Fisher's exact test. Temporal trends in subtype incidence were evaluated using binomial logistic regression, with additional models adjusting for age and sex, and subtype-specific changes expressed as odds ratios (OR) with 95% confidence intervals and average marginal effects on the probability scale (percentage-point change per year, Δpp/year). Differences between patients in or outside large clusters were assessed using binomial logistic regression for categorical traits and for continuous traits dichotomized at the population median (age, CD4+ T cell count, and viral load), while two-sided t-tests were used to compare the original continuous values. To test associations between cherry type and patient characteristics we used chi-squared tests.

For phylogenetic distance calculations, we used the normalized Robinson-Foulds distance (Robinson and Foulds, 1981), quartet divergence (Estabrook et al., 1985), and Clustering Information Distance (Smith, 2020) metrics from the TreeDist R package (Smith, 2022). Ancestral states and the gain or loss of mutations along the largest TDR clade phylogeny were inferred using maximum parsimony.

To estimate emigration from and immigration to Hungary for specific countries, we used international bilateral migration flow data between 2015 and 2020 (Abel and Cohen, 2019) and inbound/outbound tourism statistics up to 2024 from the Hungarian Central Statistical Office (https://www.ksh.hu/stadat?lang=hu&theme=tur, accessed on April 2, 2025). HIV prevalence estimates were derived from the Global Burden of Disease Study 2021 Results (Global Burden of Disease Collaborative Network, 2022). Data on the number of sequences uploaded to NCBI GenBank by country of origin were queried on April 3, 2025. We modeled Markov jump imports and exports between Hungary and other countries using a joint multivariate negative binomial regression model. Predictors included bilateral migration rates (estimated with a closed demographic accounting system and pseudo-Bayesian method, retaining the sum of immigration and emigration), national HIV prevalence per 100,000, and the ratio of published sequences to the estimated number of PLWH. Continuous predictors were log-transformed and standardized. Model selection was assessed by absolute Akaike information criterion (AIC) values, and predictor importance was evaluated using likelihood-ratio tests. Observed vs. expected counts were compared to identify countries with over- or underrepresentation in international HIV connections.

Statistical significance was defined as *P* < 0.05 (two-tailed) for all tests. All statistical tests and visualizations were performed in R v4.3.1(R Core Team, 2023).

## 3 Results

### 3.1 Characteristics of the study population

**Figure 1:**
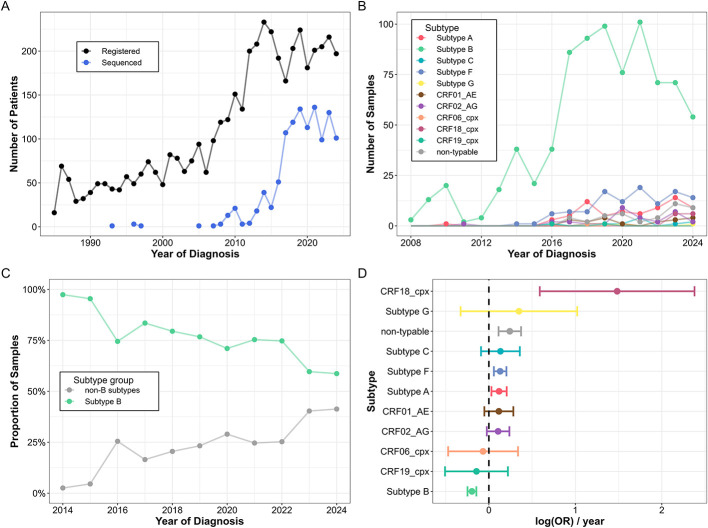
Sampling intensity and subtyping in Hungary until the end of 2024. **(A)** The number of new registered and sequenced samples per year in Hungary. **(B)** Subtype distribution of sequenced samples from Hungary by year. **(C)** The ratio of subtype B and non-B subtypes among sequences from Hungary between 2014 and 2024. **(D)** Trend analysis of HIV-1 subtype incidence in Hungary. Odds ratios (OR) for subtype detection over the study period are shown with 95% confidence intervals (error bars). Error bars are colored by subtype.

A total of 1,120 patients diagnosed with HIV-1 between 1993 and 2024 were analyzed (with samples collected between 2008 and 2024), with the majority being therapy-naïve at the time of sampling (1,063 of 1,120; 94.9%) (Figure 1A). PrEP usage within the treatment-naïve group was documented in three cases (3 of 1,063; 0.3%). Among patients with known nationality (897 of 1,120), 8.5% (76 of 897) originated from countries other than Hungary, most commonly Ukraine (*n* = 17), Brazil (*n* = 5), China (*n* = 5), Romania (*n* = 5), Russia (*n* = 5), Serbia (*n* = 4), Argentina (*n* = 2), and Italy (*n* = 2), along with 31 other countries (*n* = 1 for each). The most common route of infection was among men who have sex with men (MSM; 951 of 1,120; 84.9%), followed by heterosexual transmission (HET) in 112 (10.0%) cases, maternal transmission (MTCT) in 7 (0.6%), injection drug use in 1 case (0.1%); transmission route was unknown for 49 (4.4%) patients. The majority of patients were male (1,034; 92.3%), female patients accounted for 84 (7.5%) cases, and the sex of 2 (0.2%) patients was not reported. The median age at diagnosis of the study group was 34 years (IQR: 27–42). At the time of sample collection, the median viral load was 56,000 copies/mL (IQR: 12,400–210,000), and the median CD4+ T cell count was 418 cells/μL (IQR: 241–613). The characteristics of the study population are summarized in Table 1.

**Table 1:** Characteristics of the study population.

**Variable**	**Group**	**Total population**		**Subtype B**		**Non-B subtypes**		***P*-value**
		***n***	**%**	***n***	**%**	***n***	**%**	
Samples		1,120	100%	815	72.8%	305	27.2%	-
Sex	Male	1,034	92.3%	**772**	94.7%	**262**	85.9%	-
	Female	84	7.5%	**41**	5.0%	**43**	14.1%	**< 0.0001**
	UNK	2	0.2%	2	0.2%	0	0%	-
Age group	**I** < 20	20	1.8%	11	1.4%	9	2.9%	0.314
	**II** 20–29	364	32.5%	264	32.4%	100	32.8%	1.000
	**III** 30–39	372	33.2%	283	34.7%	89	29.2%	0.264
	**IV** 40–49	207	18.5%	144	17.7%	63	20.7%	0.426
	**V** ≥50	109	9.7%	74	9.1%	35	11.4%	0.426
	UNK	48	4.3%	39	4.8%	9	3.0%	-
Route of infection	MSM	951	84.9%	**727**	89.3%	**224**	73.4%	**0.003**
	HET	112	10.0%	**53**	6.5%	**59**	19.3%	**< 0.0001**
	MTCT	7	0.6%	1	0.1%	6	2.0%	0.27
	PWID	1	0.1%	0	0.0%	1	0.3%	-
	UNK	49	4.4%	34	4.2%	15	4.9%	-
CD4+ T cell count (cells/mm^3^)	< 200	212	18.9%	146	17.9%	66	21.6%	0.140
	≥200	824	73.6%	611	75.1%	213	69.8%	-
	UNK	84	7.5%	58	7.1%	26	8.5%	-

Non-B subtypes detected in this study: F (112; 10.0%), A (71; 6.3%), CRF02_AG (31; 2.8%), CRF01_AE (19; 1.7%), CRF18_cpx (12; 1.1%), C (11; 1.0%), G (2; 0.2%), CRF06_cpx (2, 0.2%), CRF19_cpx (2, 0.2%) and non-typable sequences (43, 3.8%). Bold text designates statistically significant difference in the prevalence of non-B subtypes between groups (Fisher's exact test was used to compare frequencies; statistical significance was defined at two-tailed *P* < 0.05). Each age group and risk group (by route of infection) was compared with the cumulative data of other groups. MSM, men who have sex with men; HET, heterosexual contact; MTCT, maternal contact; PWID, people who inject drugs; UNK, unknown.

### 3.2 Frequency and trends of HIV-1 subtypes

Using information from three subtyping tools, we found that the majority of patients carried subtype B HIV-1 strains (814; 72.7%), while pure subtypes F (112; 10.0%), A (71; 6.3%; sub-subtypes: A6: 57 and A1: 9, ambiguous: 5), C (11; 1.0%) and G (2; 0.2%), and CRFs CRF02_AG (31; 2.8%), CRF01_AE (19; 1.7%), CRF18_cpx (12; 1.1%), CRF06_cpx (2; 0.2%) and CRF19_cpx (2; 0.2%) were also detected (Figure 1B). We classified 43 sequences as non-typable due to ambiguous subtyping and considered them as “non-B” in subsequent analyses.

The annual incidence of subtype B significantly decreased over the study period, from almost complete dominance in 2014 to approximately 60% in 2024 (OR = 0.82, 95% CI 0.78–0.86; Δpp/year = −3.68; FDR < 0.0001) (Figures 1C, D). This decline in incidence remained significant after adjusting for co-factors (age and sex) in multivariate analyses (OR = 0.83, 95% CI 0.78–0.87; Δpp/year = −3.52; *P* < 0.0001) and when the test was restricted to men (OR = 0.81, 95% CI 0.77–0.86; Δpp/year = −3.66; *P* < 0.0001). The decreasing trend of subtype B was observed among MSM (OR = 0.82, 95% CI 0.77–0.87; Δpp/year = −3.42; OR < 0.0001), but for the HET transmission group it was not significant (OR = 0.92, 95% CI 0.82–1.04; Δpp/year = −2.02; *P* = 0.188) (Supplementary Figure 1). Among non-B subtypes, the incidence of subtype A (OR = 1.13, 95% CI 1.03–1.13; Δpp/year = 0.80; FDR = 0.020), subtype F (OR = 0.81, 95% CI 0.77–0.86; Δpp/year = −3.66; FDR = 0.002), CRF18_cpx (OR = 4.40, 95% CI 1.80–10.7; Δpp/year = 1.64; FDR = 0.003), and non-typable sequences (OR = 1.27, 95% CI 1.12–1.45; Δpp/year = 0.89; FDR = 0.001) increased significantly over the study period.

The ratio of non-B subtypes was significantly associated with female sex (*P* < 0.0001) and with the HET risk group (*P* < 0.0001) compared to all other risk groups (Table 1). The most prevalent non-B subtypes among HET individuals were A (18/112; 16.1%), F (15/112; 13.4%), CRF02_AG (9/112; 8.0%), CRF01_AE (7/112; 6.3%), and C (4/112; 3.6%). The percentage of females and non-MSM individuals was exceptionally high in the case of subtype C (45.5% and 54.6%), CRF01_AE (26.3% and 36.8%) and CRF02_AG (22.6% and 30.0%) and lower for more prevalent subtypes such as subtype A (12.7% females and 26.5% non-MSM), B (5.05% and 6.92%) and F (10.7% and 18.2%) and non-typable sequences (11.6% and 23.3%). The ratio of B compared to non-B subtypes showed no significant association with age groups or CD4+ T cell number at diagnosis (Table 1).

### 3.3 Maximum likelihood phylogenetic reconstruction and clustering analysis of sequences from Hungary

**Figure 2:**
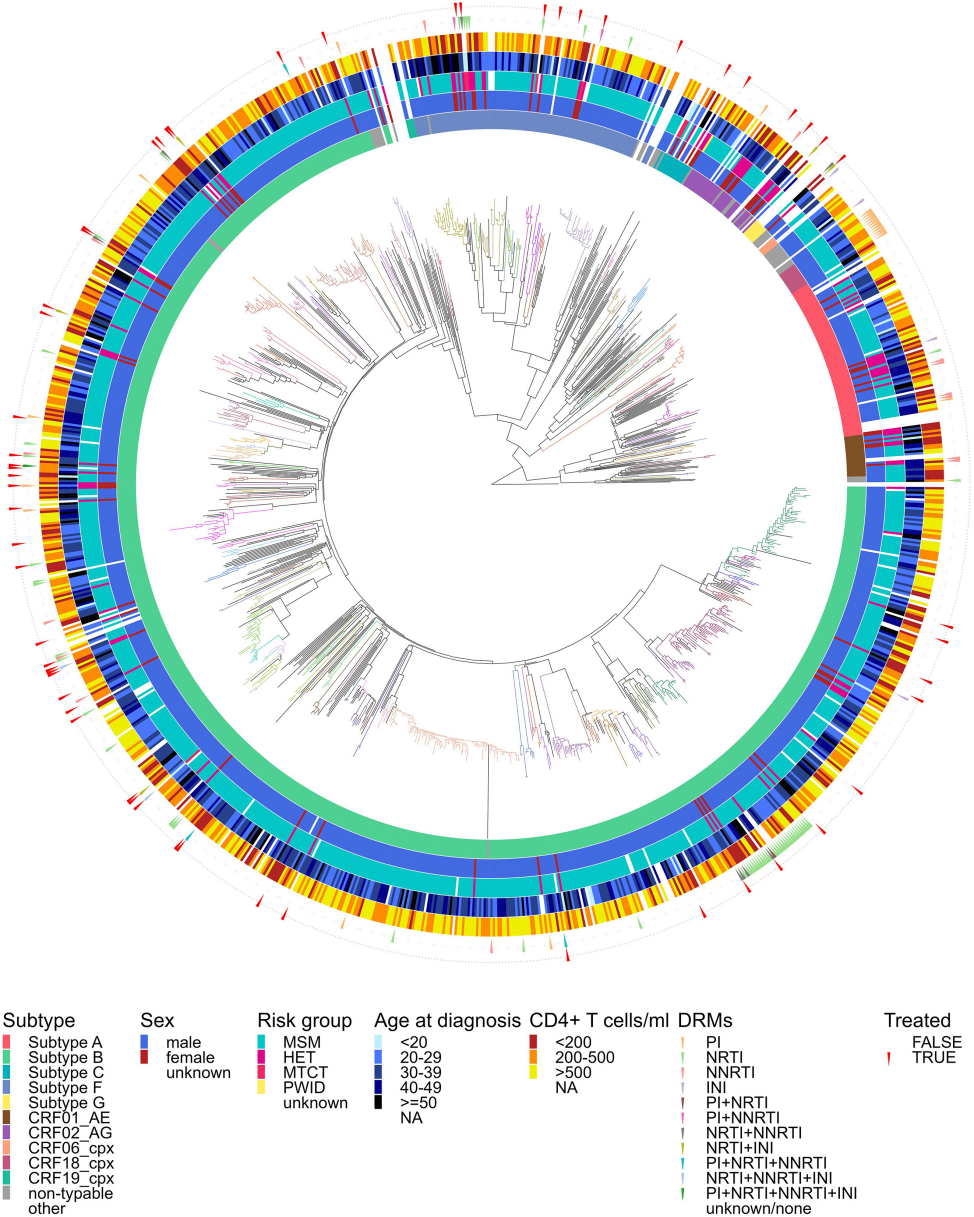
Maximum likelihood phylogenetic tree of HIV-1 sequences obtained in Hungary up to the end of 2024. A reference dataset encompassing HIV-1 group M subtypes (A, B, C, D, F, G) and circulating recombinant forms (CRFs) present in Hungary was downloaded from the Los Alamos HIV Sequence Database (RIP Alignment 2023). Branches of the phylogenetic tree are colored according to transmission clusters. Colored concentric circles around the phylogeny denote the following patient/viral characteristics corresponding to each sequence (from innermost to outermost): (1) subtype of the sequence; (2) biological sex of the patient; (3) most likely route of infection (MSM, men who have sex with men; HET, heterosexual contact; MTCT, mother to child transmission; PWID, people who inject drugs); (4) age at diagnosis, (5) CD4+ T cell count, (6) surveillance drug resistance mutations grouped by drug class (PI, protease inhibitor; NRTI, nucleoside reverse transcriptase inhibitor; NNRTI, non-nucleoside reverse transcriptase inhibitor; INI, integrase inhibitor); and (7) positive or negative antiretroviral treatment history at the time of sampling.

To characterize the domestic dynamics of the epidemic in Hungary, we first conducted analyses on the set of sequences obtained in Hungary. A total of 198 partial *pol* sequences reported in earlier studies (Mezei et al., 2011; Áy et al., 2020), along with the 922 reported in this study, were included in the phylogenetic and transmission clustering analyses, resulting in a final dataset of 1,120 sequences (Figure 2 and Supplementary Figure 2). Overall, out of the 1,120 total sequences, 979 (87.4%) included both the partial PRRT and INT regions, 139 (12.4%) included only PRRT, and 2 (0.2%) included only INT.

Our applied molecular clustering approach identified 136 possible transmission clusters, each containing at least two sequences from Hungary. Approximately two-thirds of the sequences (846/1120; 75.5%) were part of clusters. Among clustered patients from Hungary with available metadata, the majority were male (95.7%), belonged to the MSM population (92.8%), and carried subtype B strains (75.5%) (Supplementary Table 1). Of the 76 patients with a nationality other than Hungarian, 33 (43.4%) were part of clusters.

Cluster sizes ranged from 2 to 96 (average = 6.2, median = 3, IQR: 2–5), with 17 clusters containing at least 10 sequences. These large clusters included 39.5% (442/1120) of all sequences, and the patients in these clusters were more frequently MSM (93.4% vs. 79.4%; OR = 3.71 (2.47–5.74), *P* < 0.0001), younger at sampling (median 33.6 vs. 37.1 years; OR for age below the population median = 1.94 (1.52–2.47), *P* < 0.0001), and had higher CD4+ T cell counts (median 520 vs. 404 cells/mm^3^; OR for CD4 above the population median = 2.33 (1.81–3.00), *P* < 0.0001) and higher viral loads (median 4.75 vs. 4.63 log copies/mL; OR for viral load above the population median = 1.42 (1.11–1.82), *P* = 0.005) compared with patients assigned to no or to smaller clusters. Of the 17 large clusters, 12 consisted of subtype B sequences (9 patients with non-Hungarian nationality from 6 clusters), while we also detected two subtype F, one subtype A (A6, one patient with non-Hungarian nationality), one CRF02_AG (one patient with non-Hungarian nationality), and one CRF18_cpx cluster (one patient with non-Hungarian nationality) with at least ten sequences. Only one non-Hungarian patient was in a basal position (a Romanian patient within the largest cluster, *n* = 96), suggesting that all others likely acquired the infection in Hungary.

Four transmission clusters (*n* = 19) contained only non-typable sequences, all detected after 2018. An additional phylogenetic analysis with a more extended reference dataset indicated that their closest relatives were CRF56_cpx (two clusters of sizes 7 and 3), CRF20_BG (*n* = 3), and CRF141_BF (*n* = 6, including three non-Hungarian patients, one of whom—a Chinese patient—was in a basal position).

We performed sensitivity analyses for transmission cluster identification using a wide range of commonly applied parameters. The number of identified clusters and average cluster size varied considerably for ClusterPicker and HIV-TRACE when applied separately (Supplementary Figures 3A, 4). Using the union of the two methods identified 116–182 transmission clusters with an average size of 4.2–7.7, encompassing 46.0%−85.6% of all sequences (Supplementary Figure 5).

### 3.4 Transmitted drug resistance

We identified four TDR clades (including 5.0% of sequences overall) based on the clustering of surveillance drug resistance mutations (SDRMs) and polymorphisms in the Hungarian-only phylogeny (Figure 2). Three of these corresponded directly to clusters identified by our molecular clustering approach. The fourth and largest TDR clade was divided into three clusters (along with several non-clustering sequences) by the automated analysis due to the high genetic divergence within the clade (Supplementary Figure 6). Two of the 36 patients had received treatment at the time of sampling, with one occupying a basal position in the clade, suggesting that the origin of these SDRMs may have resulted from unsuccessful treatment of a patient in Hungary. The 36 sequences in this subtype B clade carried two characteristic DRMs: RT M41L (28/36) and RT T215E (36/36). Three additional polymorphisms were also prevalent: PR A71V (36/36), INT S230N (25/26), and INT M50I (10/26). The emergence of these five mutations was located near the root of the clade, and loss of mutations occurred several times, most often for RT M41L (6 times). Loss of INT M50I characterized a subclade that corresponded to the largest cluster within the clade identified by our molecular clustering approach. The combination of M41L and T215E, observed in most sequences, is associated with low to intermediate resistance to certain nucleoside reverse transcriptase inhibitors (NRTIs). In addition to the major TDR clade, a smaller subtype B cluster (*n* = 3) carried the RT T215E mutation only, which confers potential low to intermediate resistance to certain NRTIs. A subtype F cluster (*n* = 5) was characterized by the presence of PR L10V (5/5), INT S230N (5/5), RT T215S (4/5), and RT M41L (4/5), with the last two mutations contributing to low or intermediate NRTI resistance. Finally, a CRF18_cpx cluster (*n* = 12) contained PR F53Y, PR K10I, and INT L74I mutations in all sequences, none of which are associated with PR or INT resistance.

### 3.5 Cluster growth analysis

**Figure 3:**
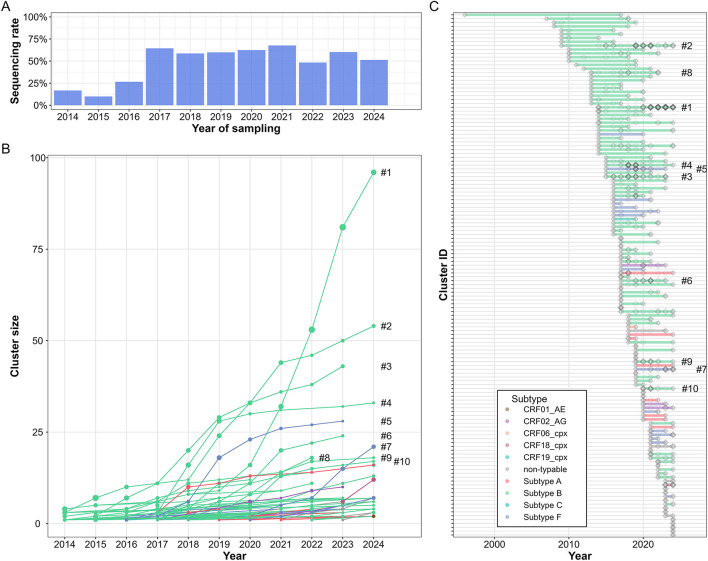
Cumulative size of clusters identified from Hungarian HIV-1 sequences up to the end of 2024. **(A)** Sequencing rate of new diagnoses in Hungary (excluding anonymous testing) since 2014. Diagnosed cases lacking identifying information in the national surveillance system were excluded from this analysis to avoid bias from potential duplicates. **(B)** Cumulative size of domestic clusters over time, where sequences were added to clusters based on the patient's diagnosis date when available; otherwise, they were added based on the sequence sampling date. The cumulative cluster size represents the total number of patients diagnosed up to a given year, with point sizes proportional to their share among all new diagnoses in that year. **(C)** Active time periods of all identified Hungarian clusters, approximated by the first and last recorded diagnoses within each cluster. Individual sequences are represented by gray diamonds, with increasing opacity indicating the number of new diagnoses in the cluster in a given year. Cluster timelines are colored according to sequence subtype. In both panels, the 10 largest clusters at the end of 2024 are labeled from 1 to 10 in descending order of size.

We analyzed the detectable growth of clusters based on the time of diagnosis for samples within each cluster (Figure 3). Using our main clustering parameters, we found that during the 2023–2024 period, 58 out of 136 clusters (42.65%) grew by at least one sequence, while 5 out of 136 clusters (3.68%) expanded by at least five sequences. Sequences added to these five clusters accounted for 35.7% (82/230) of all newly diagnosed and sequenced cases in 2023–2024.

The three largest clusters identified (designated as #1, #2, and #3 in Figure 3) were MSM-dominant (>85% MSM) subtype B clusters with first samples collected in 2014, 2009, and 2015. By the end of 2024, these clusters contained 96, 54, and 43 sequences, respectively, with 42, 8, and 5 sequences added during the 2023–2024 period. This corresponded to a factor of expansion over the 2 years of 1.78, 1.17, and 1.13, respectively, and meant also that approximately one in six new diagnoses in the last 2 years could be linked to the single largest cluster. In addition, two MSM-dominant non-B subtype clusters exhibited substantial growth during the investigated period. A subtype F cluster (#7 in Figure 3), first detected in 2019, expanded from 7 to 21 sequences between 2023 and 2024. Similarly, a CRF18_cpx cluster, first identified in 2023, rapidly grew to 12 sequences by the end of 2024.

In our sensitivity analyses, the proportion of clusters that grew by at least one sequence ranged from 30.6% to 51.6%, while the proportion of clusters expanding by at least five sequences ranged from 1.3% to 5.6% (Supplementary Figure 7).

### 3.6 Extended phylogenetic analysis with international sequences

**Figure 4:**
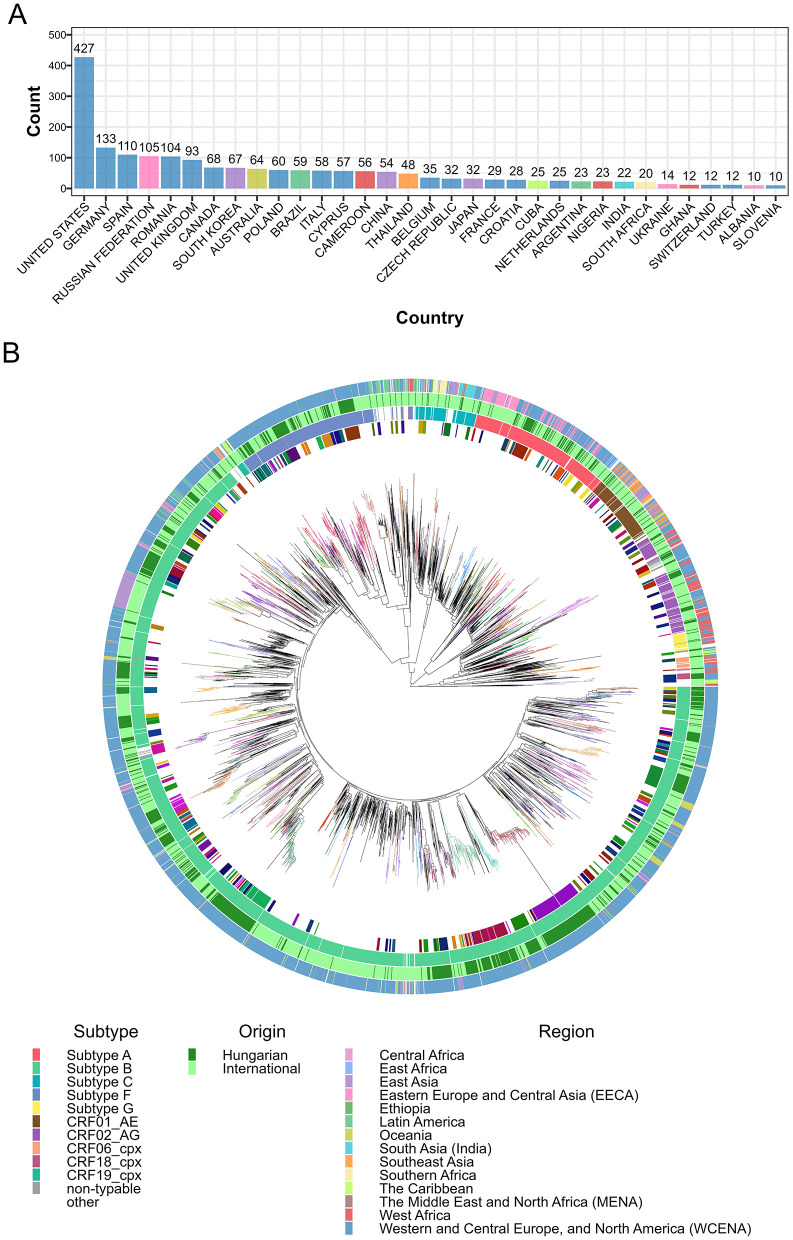
Maximum likelihood phylogenetic tree of HIV-1 sequences obtained in Hungary and the most similar international sequences. **(A)** Country of origin count data in the deduplicated set of top 5 most similar public sequences queried to isolates from Hungary. Bars representing each country are colored by geographical region, as defined by Nair et al. (2024). **(B)** Maximum likelihood phylogenetic tree of Hungarian and international background partial pol HIV-1 sequences. Branches of the phylogenetic tree are colored by transmission clusters containing at least one sequence from Hungary. Colored concentric circles denote the following patient/viral characteristics for each sequence (from innermost to outermost): (1) cluster assignment (as defined above); (2) subtype of the sequence; (3) sequence origin (Hungary or international); and (4) geographical region.

To infer the international connections of the epidemic in Hungary, we extended our maximum likelihood phylogenetic analyses by incorporating 2,202 unique international background sequences selected for the highest similarity to those reported from Hungary (Figure 4A and Supplementary Figure 1). The majority of international background sequences originated from the USA (*n* = 427), Germany (*n* = 133), Spain (*n* = 110), Russia (*n* = 105), Romania (*n* = 104), the UK (*n* = 93), Canada (*n* = 68), South Korea (*n* = 67), Australia (*n* = 64), Poland (*n* = 60), Brazil (*n* = 59), Italy (*n* = 58), Cyprus (*n* = 57), Cameroon (*n* = 56), China (*n* = 54), and Thailand (*n* = 48).

We repeated the phylogenetic clustering analysis on the extended phylogenetic tree (Figure 4B). Using the default parameters in the clustering analysis, we identified 76 Hungarian-only clusters with an average size of 6.39 sequences and 149 mixed clusters (containing at least one Hungarian and one international sequence) with an average cluster size of 7.50, including 3.05 sequences sampled in Hungary on average. The topology of larger mixed clusters (size greater than 15 and containing at least 25% sequences from Hungary) in the extended phylogenetic tree remained highly similar to their counterparts in the Hungarian-only tree, with background sequences positioned primarily at basal locations within the subtrees (Supplementary Figure 8). In our sensitivity analyses, we observed a strong negative association between the number of Hungarian and mixed clusters (Supplementary Figure 9).

**Figure 5:**
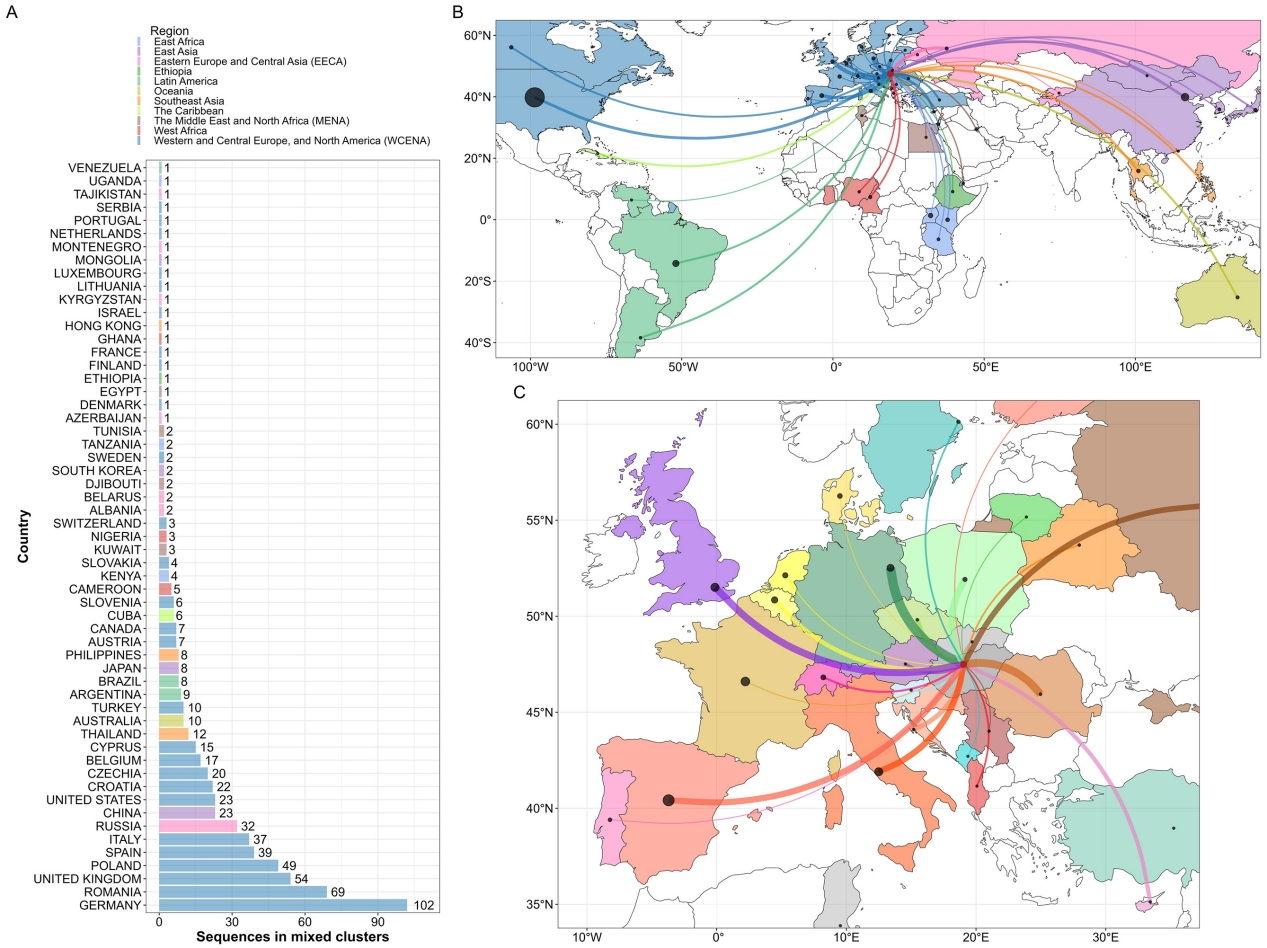
International connections of Hungarian HIV-1 sequences based on mixed sequence clusters. We identified mixed clusters (containing at least one Hungarian and one international sequence) on the maximum likelihood phylogenetic tree of Hungarian and international background sequences. **(A)** The number of sequences found in mixed clusters by country of origin. Bars representing each country are colored by geographical region, as defined by (Nair et al., 2024). Cross-border connections of Hungarian sequences within mixed clusters, displayed **(B)** worldwide, colored by geographical region and **(C)** within Europe, colored by country. The width of the lines is proportional to the number of international sequences in mixed clusters per country, while point sizes correspond to the number of sequences uploaded to the Los Alamos HIV Database from each country.

Among the background sequences included in mixed clusters, the top countries of origin were Germany (*n* = 102), Romania (*n* = 69), the UK (*n* = 54), Poland (*n* = 49), Spain (*n* = 39), Italy (*n* = 37), Russia (*n* = 32), China (*n* = 23), the USA (*n* = 23), Croatia (*n* = 22), Czechia (*n* = 20), Belgium (*n* = 17) and Cyprus (*n* = 15) (Figure 5).

The majority of background sequences in mixed clusters came from the Western and Central Europe and North America (WCENA) region (*n* = 495, subtype distribution: 61.8% B, 15.6% F, 7.68% A, 5.05% CRF02_AG, 1.98% other subtypes, and 7.89% unknown), followed by Eastern Europe and Central Asia (EECA) (*n* = 40, subtype distribution: 50.00% B, 47.50% A, and 2.50% other), East Asia (*n* = 34, subtype distribution: 35.29% B, 29.41% CRF07_BC, 20.59% CRF01_AE, 20.59% CRF02_AG, and 14.70% other), Latin America (*n* = 18, subtype distribution: 61.1% B and 38.9% other), Southeast Asia (*n* = 21, subtype distribution: 85.7% CRF01_AE and 14.3% other), Oceania (*n* = 10), West Africa (*n* = 9), the Middle East and North Africa (MENA) (*n* = 8), East Africa (*n* = 7), The Caribbean (*n* = 6) and Ethiopia (*n* = 1) regions, as defined by the modified UNAIDS geographical regions list adopted from (Nair et al., 2024) (Supplementary Figure 10).

### 3.7 Cherry and assortativity analysis

**Figure 6:**
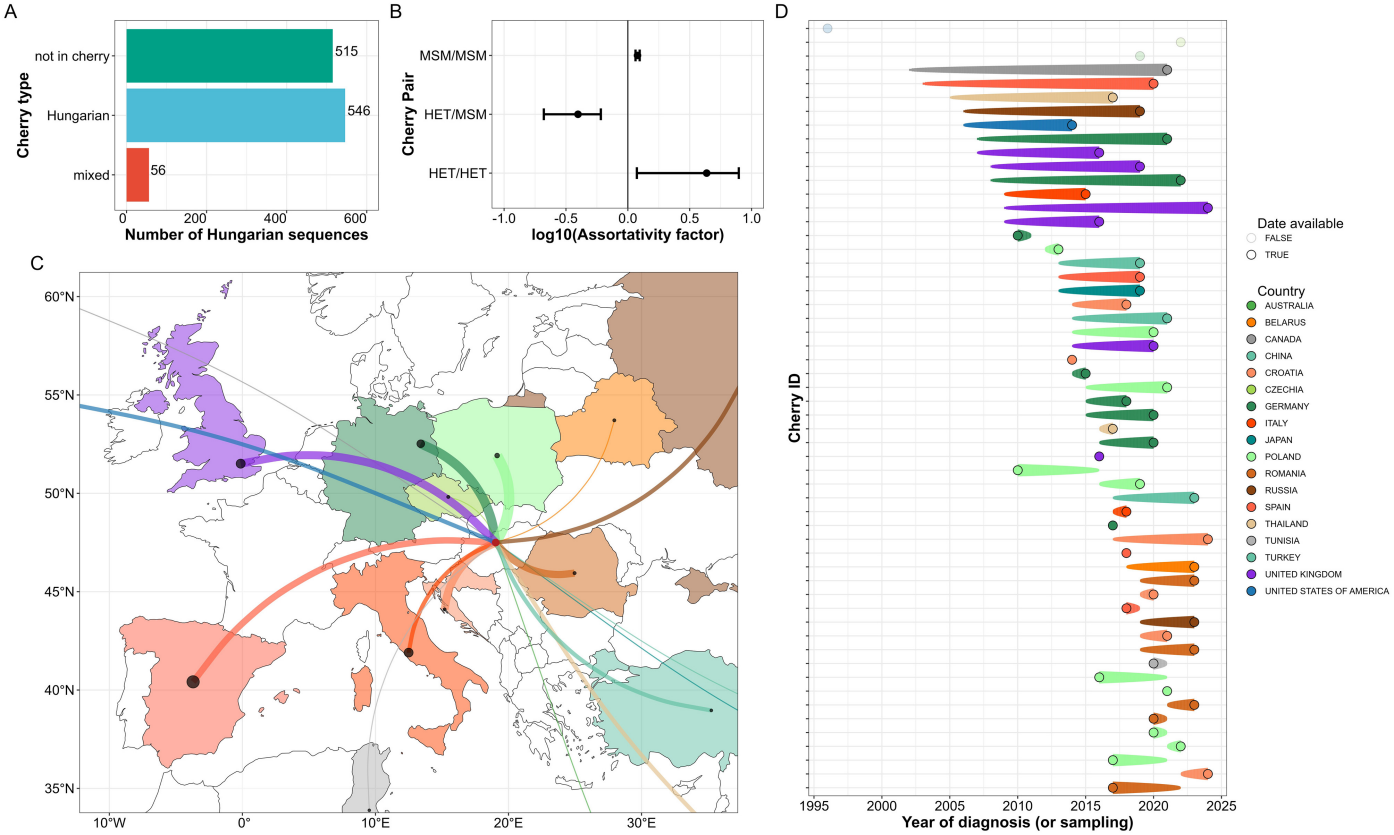
International connections of Hungarian HIV-1 sequences identified in the cherry analysis. Hungarian and mixed cherries—defined as genetically closely related monophyletic pairs containing wither two Hungarian or one Hungarian and one international sequence respectively—were identified based on a maximum cophenetic distance of 0.045. **(A)** Distribution of Hungarian HIV-1 sequences by cherry type. **(B)** Assortativity of risk groups based on domestic cherries. An assortativity factor (AF) above 1 indicates a higher frequency of cherry pairs with a given risk group combination than expected under random pairing of tips, while AF < 1 indicates overdispersion compared to random pairing. Risk group abbreviations: MSM, men who have sex with men; HET, heterosexual. Error bars indicate 95% confidence intervals. **(C)** Map of cross-border connections of Hungarian sequences in mixed cherries within Europe. The width of the lines is proportional to the number of international sequences in mixed cherries per country, while point sizes correspond to the number of sequences uploaded to the Los Alamos HIV Database by each country. **(D)** Difference in time of sample collection between the Hungarian and international sequences in mixed cherries. Cherries are colored by the country of origin of the international sequence. Cases where the international background sequence lacks a sampling date are faded out. The date of each sample (indicated by empty circles) corresponds to the patient's diagnosis date when available or otherwise to the sequence sampling date; the narrow end of the symbols indicates the sampling year of the international sequence.

To identify closely related samples with potential infection-relatedness, we extracted all monophyletic pairs [hereafter referred to as “cherries” (Kusejko et al., 2022)] that included at least one sequence originating from Hungary from the extended phylogeny (Figure 6). These cherries were classified into two groups: domestic cherries, where both sequences originated from Hungary, and mixed cherries, where one sequence was obtained from a sample from Hungary and the other was an international background sequence. In our main analysis, we considered only cherries with a maximum genetic distance of 0.045, which included 95.5% of all domestic cherries (273/286) and 50.0% of all mixed cherries (56/112) (Supplementary Figure 11). With these settings, 48.9% of all sequences from Hungary were in domestic cherries and 5.0% in mixed cherries. Individuals in the MSM risk group were significantly more likely to be part of a cherry compared to those in the HET group (54.9% vs. 42.9%, *P* = 0.021). In contrast, no significant associations were observed between being in a cherry and subtype (54.5% for subtype B vs. 53.1% for non-B, *P* = 0.732) or sex (54.7% for males vs. 44.0% for females, *P* = 0.076). Additionally, the number of mixed cherries showed no association with subtype, sex, or risk group.

To assess the degree of separation or mixture between the transmission groups in Hungary, we conducted an assortativity analysis on the domestic cherries based on the risk group of patients. Our analysis revealed that MSM/MSM (assortativity factor, AF = 1.20, 95% CI: 1.15–1.25) and HET/HET (AF = 4.34, 95% CI: 1.18–7.89) cherries were significantly overrepresented, while MSM/HET (AF = 0.40, 95% CI: 0.21–0.60) cherries were underrepresented in our phylogeny, indicating limited mixing between the two major risk groups. These results remained qualitatively robust when the analysis was repeated using maximum cophenetic distances of 0.015, 0.03, and 0.055 for domestic cherries (Supplementary Figure 12).

We also used the results of the cherry analysis to highlight possible cases of close epidemiological linkage to other countries. The international sequences in the 56 mixed cherries originated from 18 different countries, most frequently from Poland (*n* = 10, 17.9%), Germany (*n* = 8, 14.3%), Croatia (*n* = 6, 10.7%), the UK (*n* = 6, 10.7%), Romania (*n* = 5, 8.9%), and Spain (*n* = 4, 7.1%). Overall, 83.9% (47/56) of cases originated from the WCENA region. Among the 53 cases where the sampling date of the international sequence was known, the international sequence was detected earlier in 39 cases, later in 9 cases, and in the same year in 5 cases.

### 3.8 Bayesian phylogenetic analysis of Hungarian and international sequences

To estimate the number and timing of independent HIV introduction events to Hungary, we performed a subtype-specific Bayesian phylogenetic analysis of Hungarian and international sequence sets (Supplementary Figures 13–20). The subtype F Bayesian MCC tree showed the closest agreement with the maximum likelihood consensus tree (normalized Clustering Information Distance, nCID = 0.171), followed by CRF18_cpx (nCID = 0.188), CRF56_cpx (nCID = 0.205), subtype C (nCID = 0.298), subtype A (nCID = 0.323), CRF02_AG (nCID = 0.354), subtype B (nCID = 0.370), and CRF01_AE (nCID = 0.498) (Supplementary Figure 21). In all cases, the Bayesian MCC trees showed greater similarity to the maximum likelihood consensus tree than either that expected from random trees of equal size or the average similarity among maximum likelihood bootstrap replicates. We attempted Bayesian phylogenetic reconstruction for the non-typable clusters with probable subtypes CRF56_cpx, CRF20_BG, and CRF141_BF; however, only the CRF56_cpx tree converged successfully.

**Figure 7:**
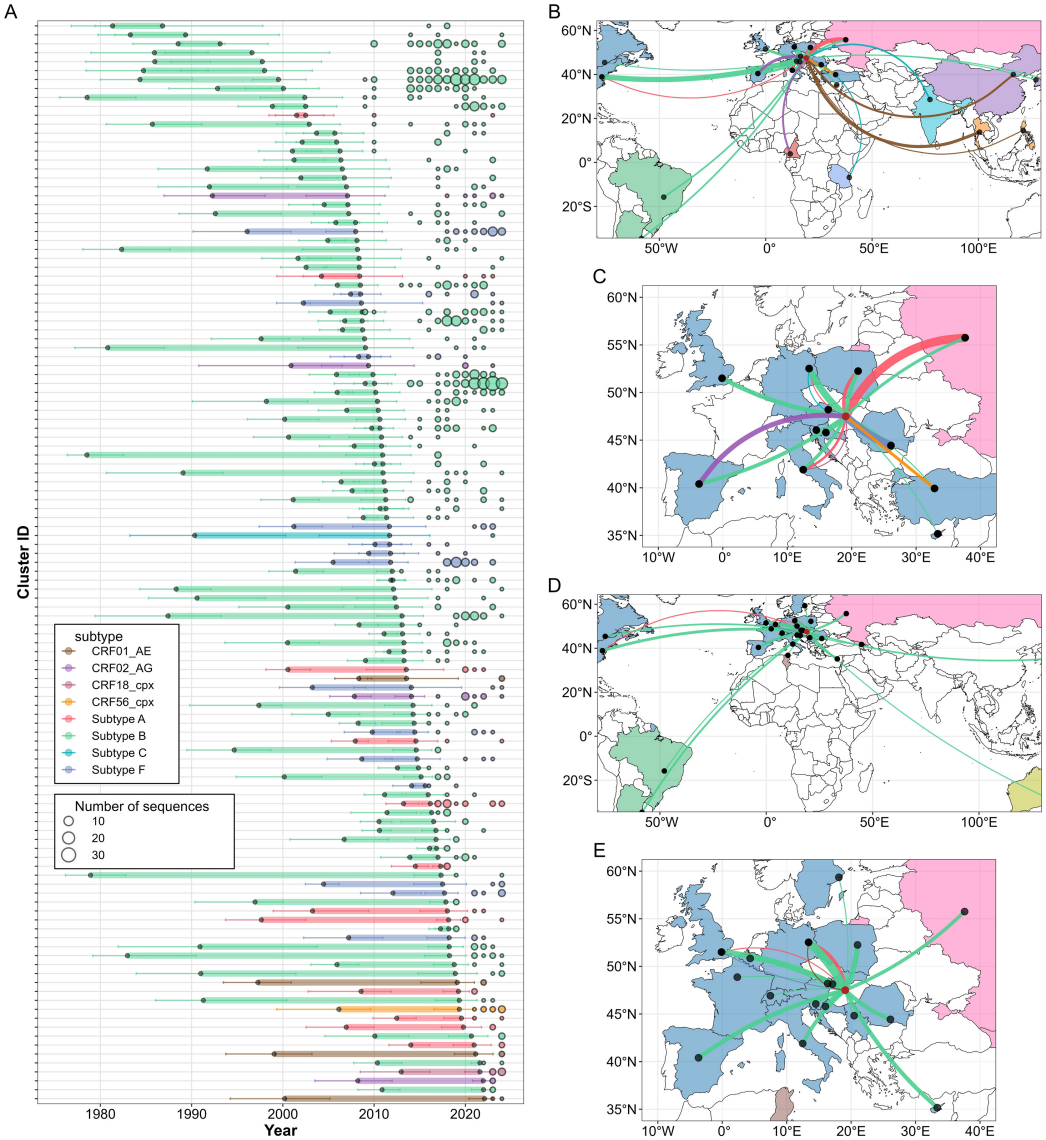
Bayesian phylogenetic and phylogeographic analyses of HIV-1 sequences obtained in Hungary and the most similar international sequences. **(A)** Estimated introduction date intervals for all lineages introduced to Hungary, defined as the time between the most recent common ancestor of sequences from Hungary and the most recent common ancestor of those sequences together with their closest international relatives. Error bars indicate 95% highest posterior density intervals. Colored circles represent the annual number of diagnosed or sampled cases from Hungary in each lineage. Lineage intervals, error bars, and circles are colored according to sequence subtype. **(B–E)** Cross-border HIV-1 import **(B)** global, **(C)** Europe and export **(D)** global, **(E)** Europe events inferred from the Bayesian phylogeographic analyses. Line widths reflect the median estimated Markov jumps per country, line colors indicate HIV-1 subtypes, and countries are shaded by geographical region.

We estimated at least 122 independent introduction events to Hungary; 83 for subtype B (time to most recent common ancestor intervals: 1979–2022), 15 for subtype F (1996–2018), 12 for subtype A (1998–2021), four for CRF01_AE (1997–2022), four for CRF02_AG (1992–2022), two for CRF56_cpx (1997–2020), one for CRF18_cpx (2013–2021) and one for subtype C (1990–2012) (Figure 7). Among the 17 large transmission clusters (≥10 sequences) identified on the Hungarian-only maximum likelihood tree, 15 corresponded to a single introduction event in the subtype-specific Bayesian MCC trees. The clades corresponding to the four active transmission clusters among the 10 largest clusters—the three subtype B clusters and the subtype F cluster described in the *Cluster growth analysis* subsection—were estimated to have been introduced between 2009–2010, 1984–1999, 2006–2017, and 1996–2007, respectively. In contrast, the six clusters inactive in 2023–24 (clusters #4, #5, #6, #8, #9, and #10 in Figure 3) were estimated to have been introduced between 2006–2008, 2005–2011, 2005–2009, 1987–2013, 2005–2008, and 1998–2002. Regarding three TDR clusters—the large subtype B clade and the small subtype B and subtype F clusters described in the *Transmitted drug resistance subsection*—introduction to Hungary was estimated between 1988–1993, 1978–2010, and 2001–2011.

### 3.9 Bayesian phylogeographic analysis

To quantify the number of import and export events from and to specific countries for each subtype, we performed Bayesian discrete phylogeographic analyses with an asymmetric substitution model. For subtype B, the most strongly supported sources of importation were the USA [median number of Markov jumps, 95% highest posterior density (HPD): 46 (35–57)], Germany [19 (14–25)], the UK [7 (4–10)], Poland [6 (4–8)], Spain [5 (2–8)], Italy [4 (2–6)], Croatia [3 (1–4)], and Russia [3 (2–5)]. Export events of subtype B were also supported, with the largest numbers inferred toward Germany [29 (22–35)], the United Kingdom [13 (6–18)], Poland [10 (7–12)], the USA[9 (3–14)], Croatia [8 (6–10)], Spain [7 (4–12)], Cyprus [6 (4–9)], Romania [6 (3–7)], Italy [5 (2–9)], Russia [4 (1–6)], Argentina [3 (1–5)], Belgium [3 (1–6)], and Czechia [3 (1–4)]. Among non-B subtypes, subtype A was estimated to have been introduced primarily from Russia [29 (19–36)] and Poland [4 (2–10)], with exports mainly directed toward Germany [5 (0–7)]. Other non-B subtypes were inferred to be predominantly imported without strong evidence of onward export, including subtype F from Romania [28 (24–32)], CRF01_AE mainly from Thailand [11 (0–15)] and China [4 (0–10)], CRF02_AG from Spain [9 (3–16)] and Cameroon [3 (0–9)], CRF18_cpx from Italy [1 (0–1)], CRF56_cpx from Turkey [3 (0–4)] and subtype C possibly from India [2 (0–5)], Germany [1 (0–4)], or Tanzania [1 (0–5)].

We compared observed Markov jump imports and exports between Hungary and other countries against model-based expectations. Expected values were derived from a multivariable joint negative binomial model using bilateral migration or tourism estimates, national HIV prevalence rates, and the ratio of published sequences to estimated PLWH (sequencing intensity) as predictors. The migration-based model provided a better fit to the observed Markov jump patterns than a model using bilateral tourism data (AIC: 23.3 vs. 34.4). Within the migration-based model, migration rate was the strongest predictor of Markov jump counts (likelihood ratio [LR] = 93.10, *P* = 0.001), followed by the ratio of published sequences to estimated PLWH (LR = 20.03, *P* = 0.001) and national HIV prevalence (LR = 13.22, *P* = 0.002). Compared to model expectations from the best fit model, the countries most overrepresented in estimated Markov jump imports were the USA (observed = 47 vs. expected = 10), Russia (32 vs. 8), Thailand (11 vs. 2), Poland (10 vs. 3), Romania (29 vs. 22) and Spain (14 vs. 8), while the most underrepresented were Ukraine (0 vs. 16), Germany (21 vs. 33), South Africa (0 vs. 12), Canada (1 vs. 12), Austria (1 vs. 11), Italy (7 vs. 16), Australia (0 vs. 8), Switzerland (0 vs. 6), and Serbia (0 vs. 6). For Markov jump exports, the most overrepresented countries were Germany (35 vs. 17), the UK (15 vs. 5), Poland (10 vs. 3), the USA (10 vs. 3), and Spain (7 vs. 3), whereas Serbia (1 vs. 17), Romania (6 vs. 13), Australia (1 vs. 7), and Ukraine (0 vs. 5) were the most underrepresented.

## 4 Discussion

Building on previous efforts to assess the prevalence and transmission of drug resistance mutations (Mezei et al., 2011; Áy et al., 2020, 2021), this study is the first to present a comprehensive molecular epidemiological analysis of the HIV-1 epidemic in Hungary. Our results offer insights into the shifting patterns and composition of the epidemic, which could serve as a good basis for targeted intervention strategies and may hold clues to the future trends of HIV-1 in other countries, as well.

The overrepresentation of the MSM exposure group and of younger age in large transmission clusters reinforces the notion that active local transmission in Hungary has been primarily driven by sexual contacts among young and middle-aged MSM. This finding is consistent with clinical observations and with our previous report, which included cases diagnosed between 2013 and 2017 in Hungary (Áy et al., 2020). Additionally, the presence of large clusters of closely related sequences and the short intervals between most diagnoses within clusters imply intense episodic transmission among MSM, driving the local epidemic similarly to observations from several other countries (Lewis et al., 2008; Sullivan et al., 2009; van Griensven et al., 2009; Phillips et al., 2013). However, while the transmission of HIV remains apparently more intense among MSM in Hungary, the higher CD4+ T cell counts at diagnosis in large clusters suggest early presentation for testing and treatment, possibly due to greater awareness of exposure risk in this group (Mukolo et al., 2013). This suggests that although individuals in sexually active MSM communities tend to present earlier for testing and treatment, further reduction of new transmissions would require even earlier diagnosis and expanded testing efforts, as already shown for both MSM and PWID (Marzel et al., 2016; Ratmann et al., 2016; Günthard et al., 2025). Our observation that individuals in larger clusters in Hungary tend to have higher viral loads is consistent with other reports (Quinn et al., 2000; Aldous et al., 2012; Wertheim et al., 2019). This phenomenon implies the possibility that HIV-1 may be undergoing natural selection for increased infectivity and virulence (Herbeck et al., 2012; Wertheim et al., 2019).

In agreement with earlier observations from other countries (Esbjörnsson et al., 2016; Kusejko et al., 2022; Nduva et al., 2022), our assortativity analysis suggests that transmission networks among MSM and HET are largely, but not completely separated, indicating a bridge role for bisexual individuals and occasional spillover between the two groups. We note that misclassification of MSM transmission based on self-reporting (Goldstein et al., 2015) may have introduced false MSM-HET cherries: in 53 out of 56 mixed MSM-HET cherries, the HET node was also male, which is suggestive of a reporting bias, and implies that the true degree of separation is likely stronger than our estimate. Nonetheless, the presence of occasional connections between HET and MSM risk groups is further supported by our observation of several transmission clusters with mixed sex and risk group composition.

Our results strongly suggest that the emergence of local transmission networks appears to be fueled by both domestic transmissions and repeated viral introductions from abroad, contributing to the increasing genetic diversity of HIV-1 and the decreasing incidence of previously dominant subtype B in Hungary. Substantial increase was observed in the incidence of subtypes A and F, CRF18_cpx and non-typable isolates (possibly belonging to or closely related to CRFs 56_cpx, 20_BG and 141_BF based on their phylogenetic position). While this rise in genetic diversity in recent years is not broadly observed across the Western and Central Europe and North America region (Nair et al., 2024), it is characteristic of several national epidemics in Central Europe, including those in Croatia (Planinić et al., 2023), Slovakia (Habekova et al., 2010), and Poland (Serwin et al., 2021). These increasingly prevalent non-B subtypes—particularly those uncommon in Europe and Hungary, such as subtype C, CRF01_AE, and CRF02_AG—were associated with female sex and with heterosexual and maternal transmission, suggesting a potential role for immigration from regions with higher rates of heterosexual transmission. This hypothesis is further supported by the observation that approximately one-third of all new HIV diagnoses in 2022 and 2023 were among individuals whose country of origin was outside Hungary. These included regions with a higher proportion of heterosexual cases, such as Central and Eastern Europe (18.3% of diagnoses in 2022 and 13.2% in 2023, respectively), Latin America and the Caribbean (3.6% and 5.3%), and South and Southeast Asia (1.3% and 3.1%), along with a notable proportion of cases with unknown origin (8.0% and 11.4%) (European Centre for Disease Prevention Control, 2024).

Regardless of the route of infection, Bayesian phylogeographic analysis revealed that subtype B introductions were mainly associated with the USA and European countries such as Germany, the UK, Poland, Spain, Italy, Croatia and Russia, while subtype A6 appeared to originate primarily from Russia and Poland; A1 from Italy; subtype F from Romania; CRF01_AE from Thailand and China; CRF02_AG from Spain and Cameroon, CRF18_cpx from Italy and CRF56_cpx from Turkey. Multiple potential export events from Hungary were also inferred, including subtype B to Germany, the UK, Poland, the USA, Croatia, Spain, Cyprus, Romania, and Italy, and subtype A6 to Germany. We showed that suspected HIV-1 import and export events—based on the number of international sequences in mixed clusters, mixed cherries, or Markov jumps—can be partially explained by known migration patterns, HIV prevalence and public sequence availability. Migration appears to account for international connections more effectively than tourism data, suggesting that long-term migration plays a more significant role than tourism (with the possible exception of the dispersal of CRF01_AE sequences from Thailand related to sex tourism (Angelis et al., 2015), which may underline the origin of the eight CRF01_AE sequences from Thailand observed in our mixed clusters).

We found that the USA, Germany, the UK, Poland, Romania, Russia, Thailand, and Spain were overrepresented in international sequence connections compared to the expectations derived from our statistical model, which incorporated international migration, HIV prevalence, and sequencing coverage data. This suggests either the presence of additional, unexplained factors driving higher transmission between vulnerable populations in Hungary and these countries, or a relative overrepresentation of publicly available sequence data from these regions that was not fully captured by our model. In contrast, we observed the complete absence of connections with some countries where links would have been expected, particularly in Hungary's close geographical vicinity—such as Ukraine (17 patients registered in the Hungarian surveillance system, of which only one heterosexual case clustered with two other Hungarian sequences), Austria, and Serbia (2 of 4 patients clustering with Hungarian sequences). This may reflect a strong underrepresentation of these countries in public sequence databases that our model may not have been able to account for, or it may indicate genuinely weaker epidemiological connections between Hungary and these neighboring countries.

Although several war refugees from Ukraine have been registered in the Hungarian HIV surveillance system since 2022, our Bayesian phylogeographic analysis found no evidence of international transmission between the two countries through the end of 2024—unlike in other European countries such as Germany (Hanke et al., 2024) and Poland (Serwin et al., 2023, 2024). Several possible explanations may account for this observation. First, while individuals living with HIV-1 from Ukraine are recorded in the Hungarian surveillance system, many may be transiting through the country without significantly contributing to the local epidemic. This is particularly plausible given that most Ukrainian patients entering the Hungarian system were already receiving treatment. Second, the limited availability of HIV-1 sequence data from Ukraine likely contributes to underdetection. Finally, delays between infection and diagnosis may have prevented our sequence-based analysis from capturing transmission events within the study period.

Our work is subject to limitations. First, the limited availability of publicly accessible HIV-1 sequence data constrained our ability to detect all potential international connections to sequences from Hungary, particularly for countries with sparse representation. Because our national dataset is more up-to-date than international repositories, potential importation events are likely to be captured more completely than export events, which may be missing from databases due to delayed reporting. Second, short partial *pol* sequences (~2,000 bp) used for routine drug resistance genotyping limit phylogenetic resolution and frequently produce low branch support values, particularly at deep nodes—as observed in our dataset (Supplementary Figure 1). Under these conditions, phylogeny-based methods that rely on maximum pairwise genetic distance and branch support thresholds (e.g., ClusterPicker) may fail to detect transmission clusters or may subdivide them, especially when monophyletic clades contain outlier sequences with greater genetic distances (Supplementary Figure 3). By integrating distance-based (HIV-TRACE) and phylogenetic (ClusterPicker) approaches, we recovered several large, highly active clusters that ClusterPicker alone either missed or subdivided (Supplementary Figure 3). The observed low quartet divergence between our subtype-specific maximum likelihood and Bayesian phylogenies indicates strong local topological concordance, and thus confirms that, despite limited resolution at deep nodes, closely related sequences were consistently placed in close topological proximity across phylogenetic methods (Supplementary Figure 21). Finally, considering that molecular clustering is sensitive to parameter choice, genetic distance and branch support parameters must be selected in accordance with the objectives of the study (Hassan et al., 2017; Novitsky et al., 2020). To assess long-term HIV-1 transmission dynamics in Hungary, we followed recommendations for the routine tracking of transmission clusters over extended periods (Hassan et al., 2017; Novitsky et al., 2020; Labarile et al., 2023) to select our default parameters. Testing a wide range of key parameters showed that overall conclusions remained consistent. Our default parameter selection is supported by the finding that 15 of 17 large clusters corresponded to a single introduction event in Bayesian analyses. Moreover, three of four TDR clades identified by the clustering of drug resistance mutations were identical to those detected by our molecular clustering approach (in which we excluded DRM positions). The remaining TDR clade shows high internal divergence, consistent with long-term persistence [diagnoses span 24 years, with the older cases previously reported in (Áy et al., 2020)], and a bootstrap support of 95 at the root node is strongly suggestive of a single transmission cluster (Supplementary Figure 6).

## 5 Conclusion

Overall, our analyses indicate that the HIV-1 epidemic in Hungary is primarily sustained by domestic transmission, characterized by a limited number of highly active transmission clusters, and complemented by repeated introductions of viral strains from abroad that subsequently initiate local spread. While the epidemic exhibits the strongest international links with Western and Central Europe, its increasing genetic diversity appears to be driven primarily by the repeated introduction and local transmission of non-B subtypes, predominantly originating from Eastern Europe as well as Southeast and East Asia. Further surveillance is needed to monitor the spread of these non-B subtypes within Hungary and into Western and other Central European countries, toward which Hungary may act as a bridge through the epidemic links that were demonstrated in our analyses.

## Data Availability

The datasets presented in this study can be found in online repositories. The names of the repository/repositories and accession number(s) can be found below: https://www.ncbi.nlm.nih.gov/genbank/, JF419394–JF419453, KX999940–KX999999, KY021932–KY021991, KY950415–KY950436, KY967234–KY967255, MK213272–MK213318, MK236491–MK236537, MK250657–MK250695, PP333487–PP333522, PP313557, PV816828–PV816852, PV816854–PV816951, PV816953–PV817030, PV817032–PV817114, PV817116–PV817176, PV817178–PV817198, PV817200–PV817202, PV817204–PV817207, PV817209–PV817298, PV817300–PV817315, PV817317–PV817339, PV817341–PV817403, PV817405–PV817416, PV817418–PV817493, PV817495–PV817508, PV817510–PV817530, PV817532–PV817552, PV817555–PV817578, PV817580–PV817592, PV817594–PV817693, PV817695–PV817697, PV817699–PV817714, PV817716–PV817727 and PV817729–PV817735 for the PR and RT regions and PP313558–PP313598, PV903188–PV903273, PV903275–PV903401, PV903403–PV903478, PV903480–PV903563, PV903565–PV903625, PV903627–PV903647, PV903649–PV903651, PV903653–PV903656, PV903658–PV903747, PV903749–PV903764, PV903766–PV903779, PV903781–PV903789, PV903791–PV903853, PV903855–PV903866, PV903868–PV903878, PV903880–PV903913, PV903915–PV903926, PV903928–PV903948, PV903951–PV903971, PV903974–PV903997, PV903999–PV904011, PV904013–PV904111, PV904113–PV904131, PV904133–PV904144, and PV904146–PV904152 for the INT region.
