# Supplementary figures and images for: Molecular epidemiology of HIV-1 in Hungary: an evolving contact zone of colliding virus subtypes
Source: Front Microbiol. 2025 Dec 16;16:1732254. doi: 10.3389/fmicb.2025.1732254 (PMC12748157; doi:10.3389/fmicb.2025.1732254)

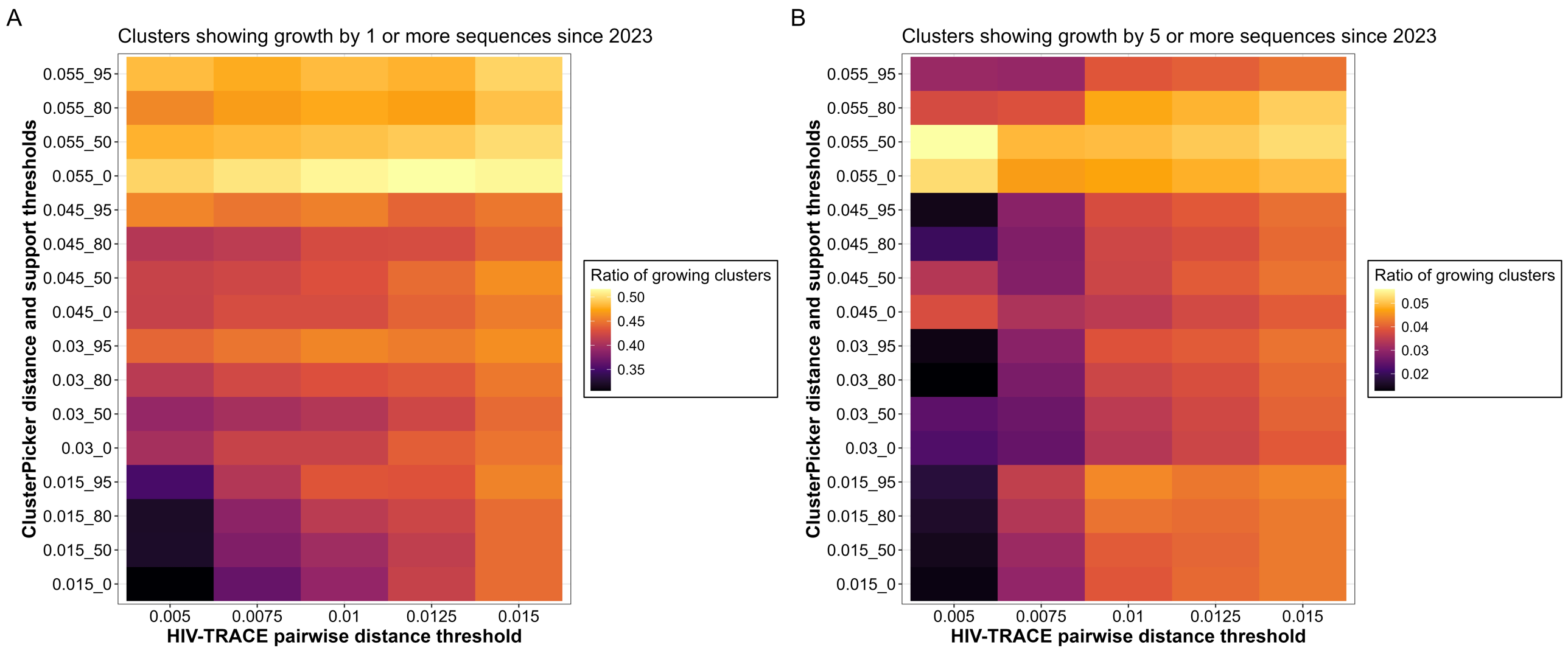

Supplement: Supplementary file 1 [file Data_Sheet_1.zip › HIVHUN_SFigure7300.jpg]

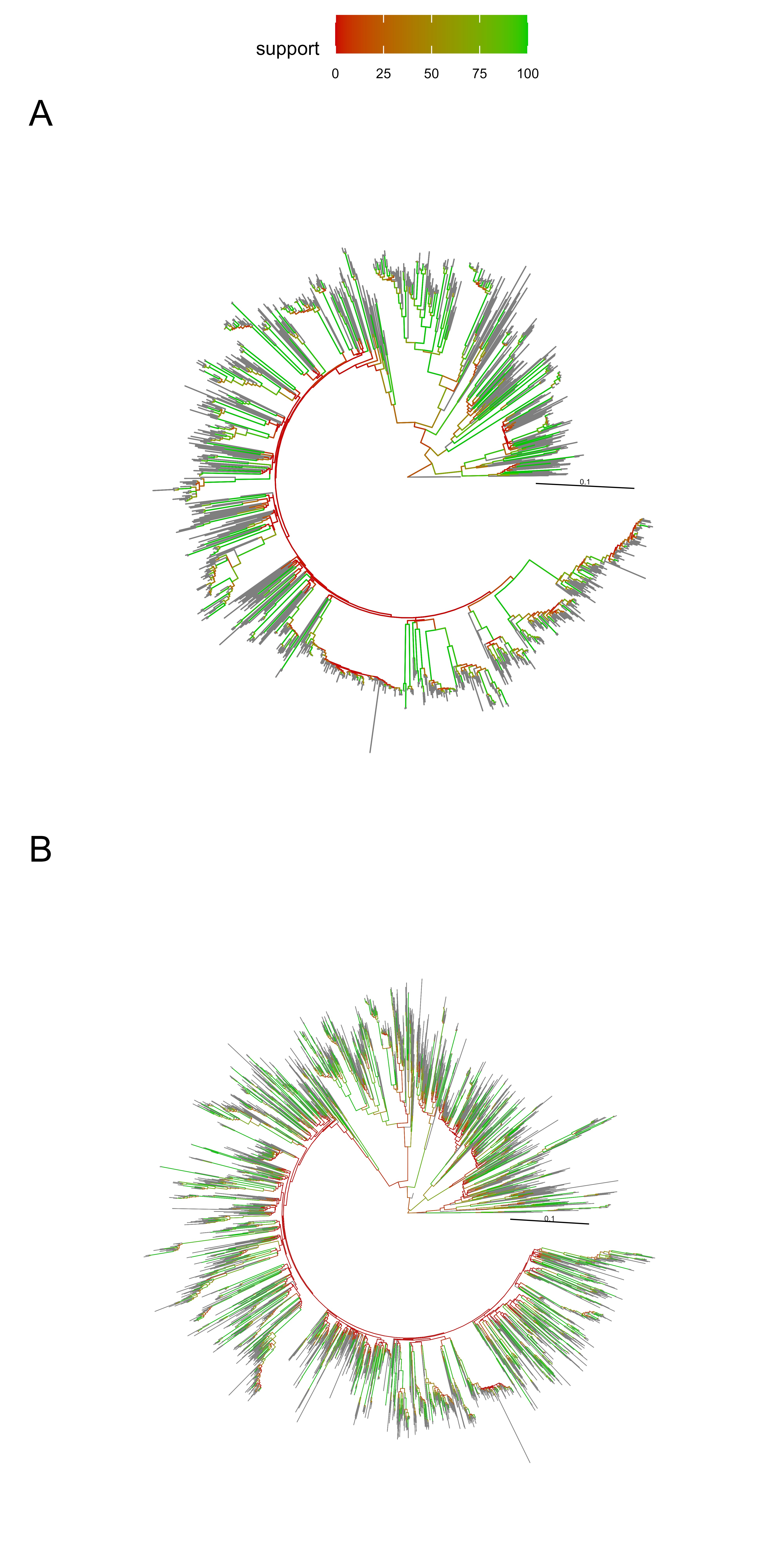

Supplement: Supplementary file 1 [file Data_Sheet_1.zip › HIVHUN_SFigure2300.jpg]

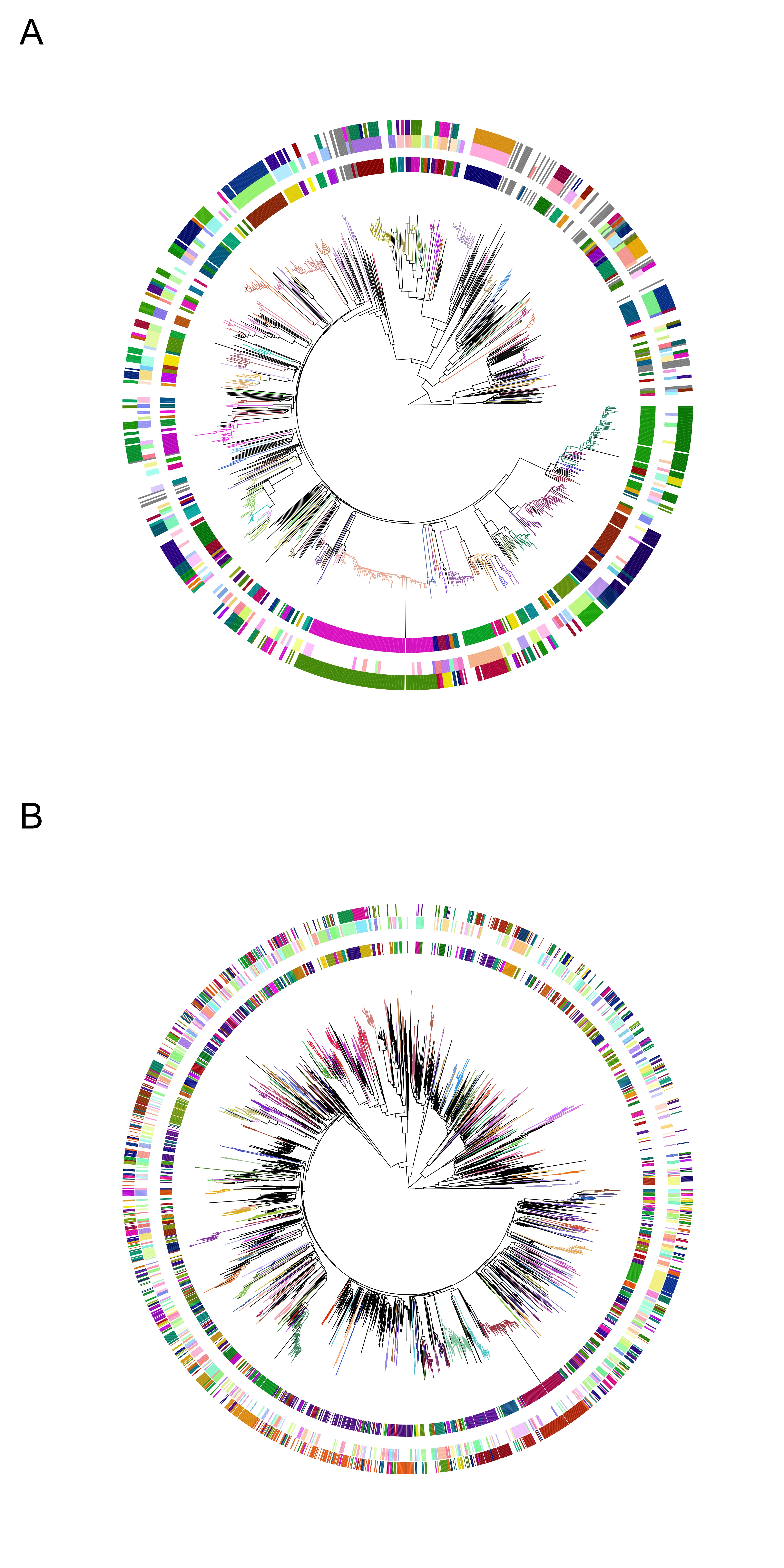

Supplement: Supplementary file 1 [file Data_Sheet_1.zip › HIVHUN_SFigure3300.jpg]

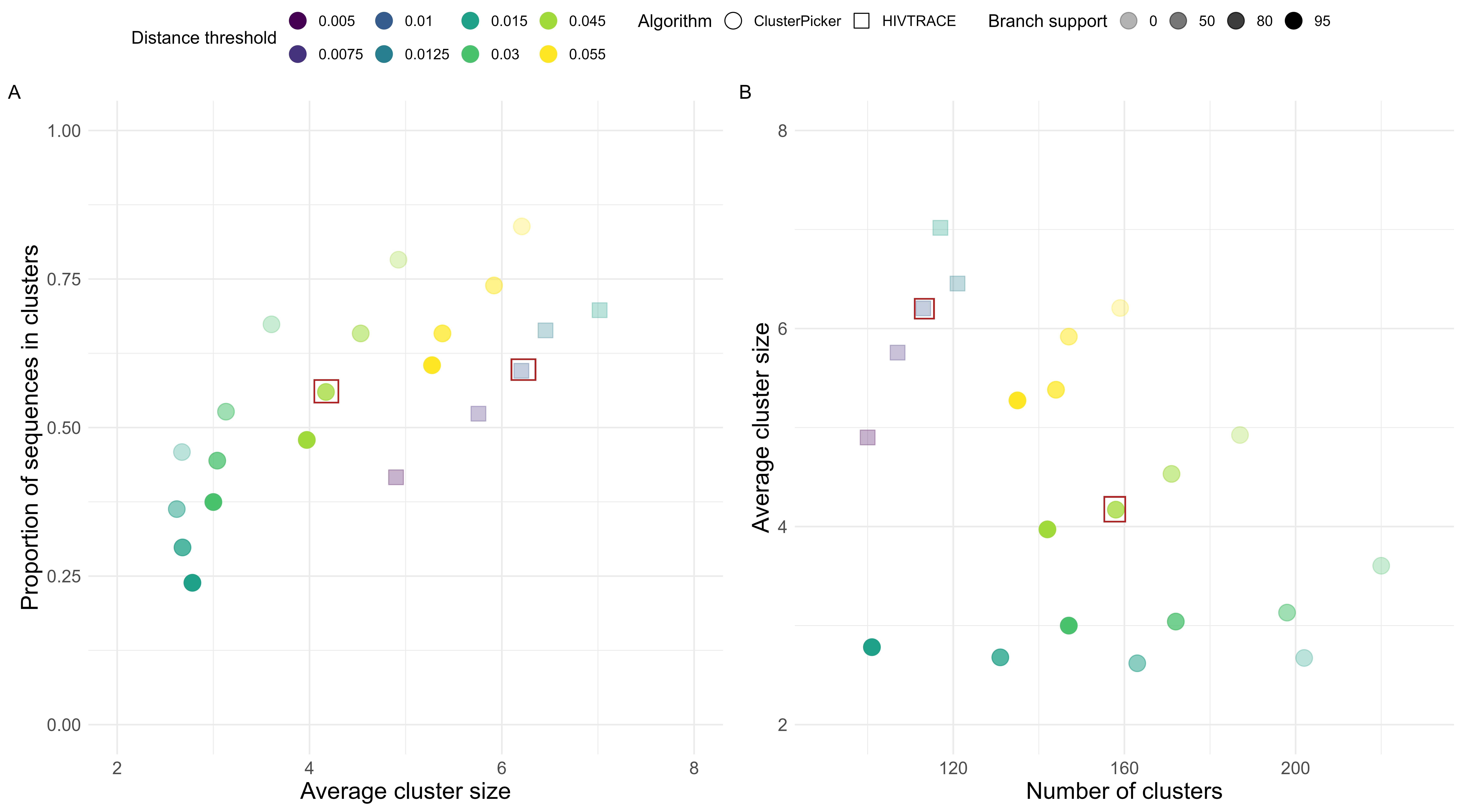

Supplement: Supplementary file 1 [file Data_Sheet_1.zip › HIVHUN_SFigure4300.jpg]

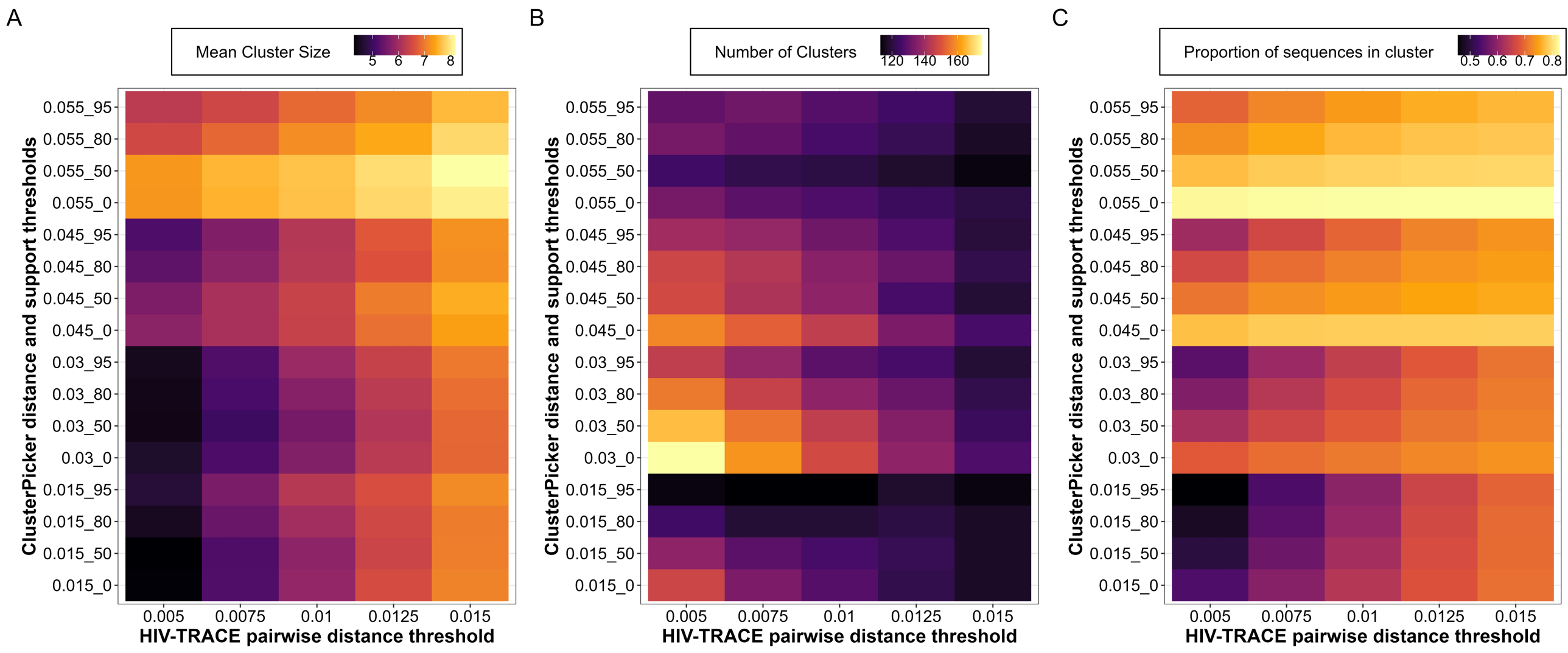

Supplement: Supplementary file 1 [file Data_Sheet_1.zip › HIVHUN_SFigure5300.jpg]

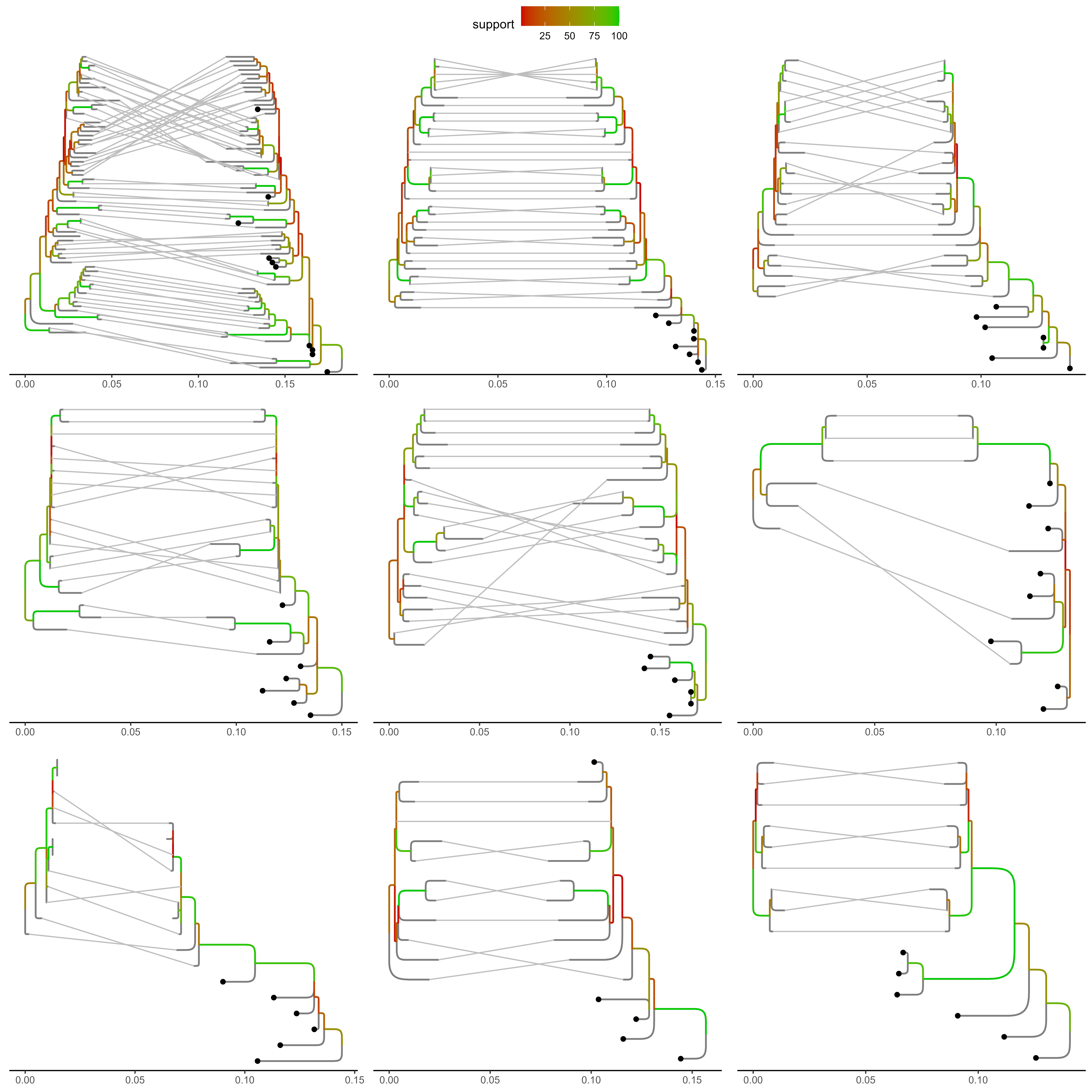

Supplement: Supplementary file 2 [file Data_Sheet_2.zip › HIVHUN_SFigure8300.jpg]

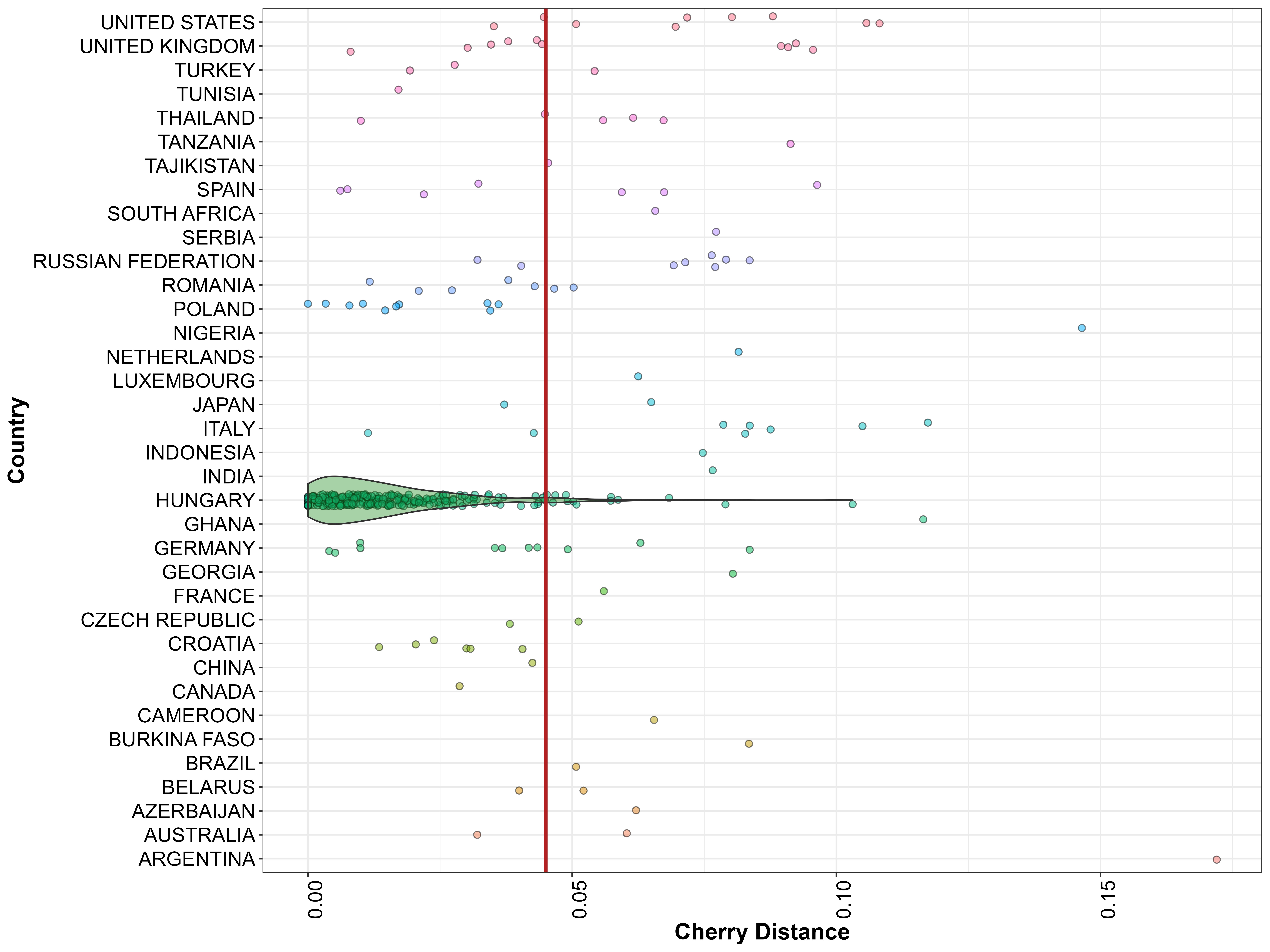

Supplement: Supplementary file 2 [file Data_Sheet_2.zip › HIVHUN_SFigure11300.jpg]

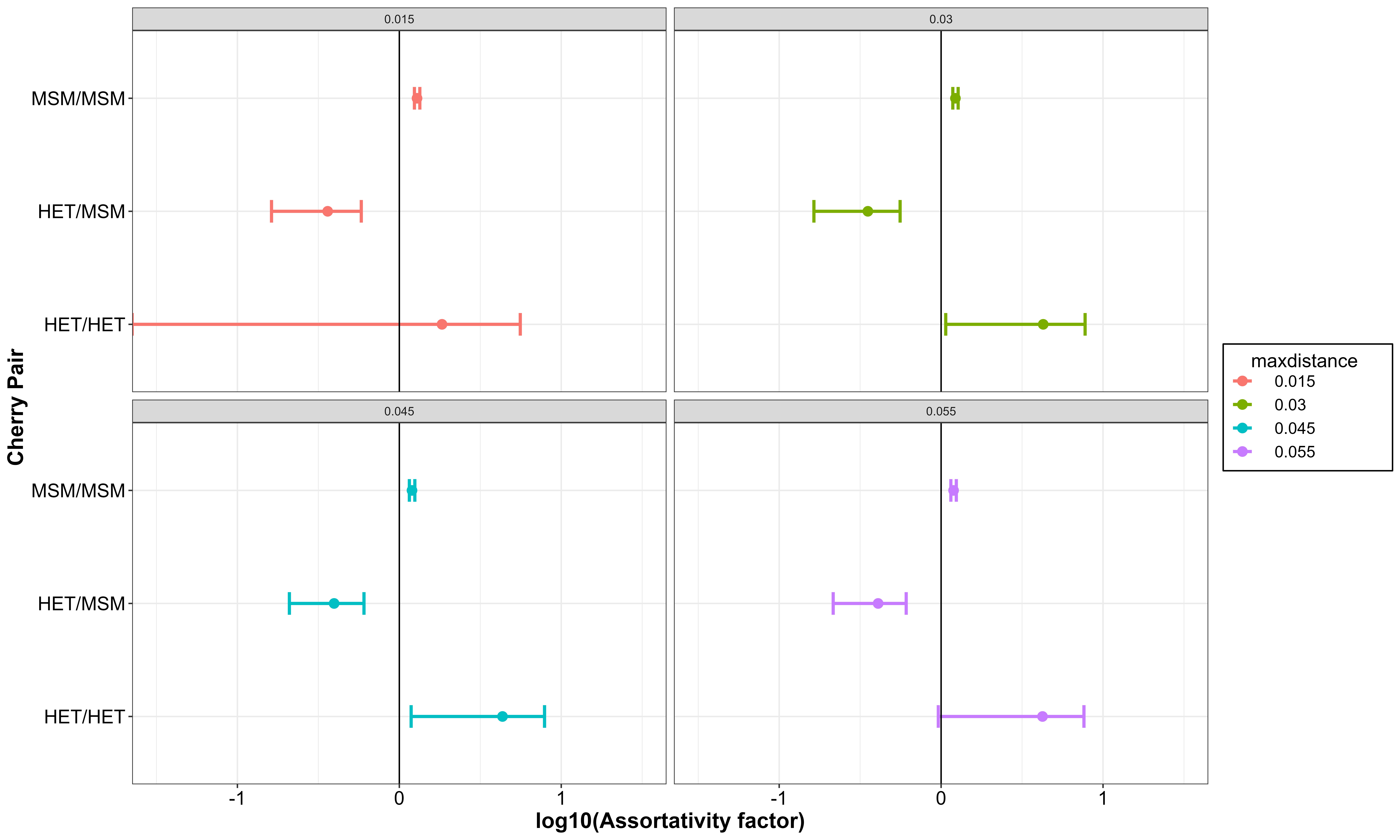

Supplement: Supplementary file 2 [file Data_Sheet_2.zip › HIVHUN_SFigure12300.jpg]

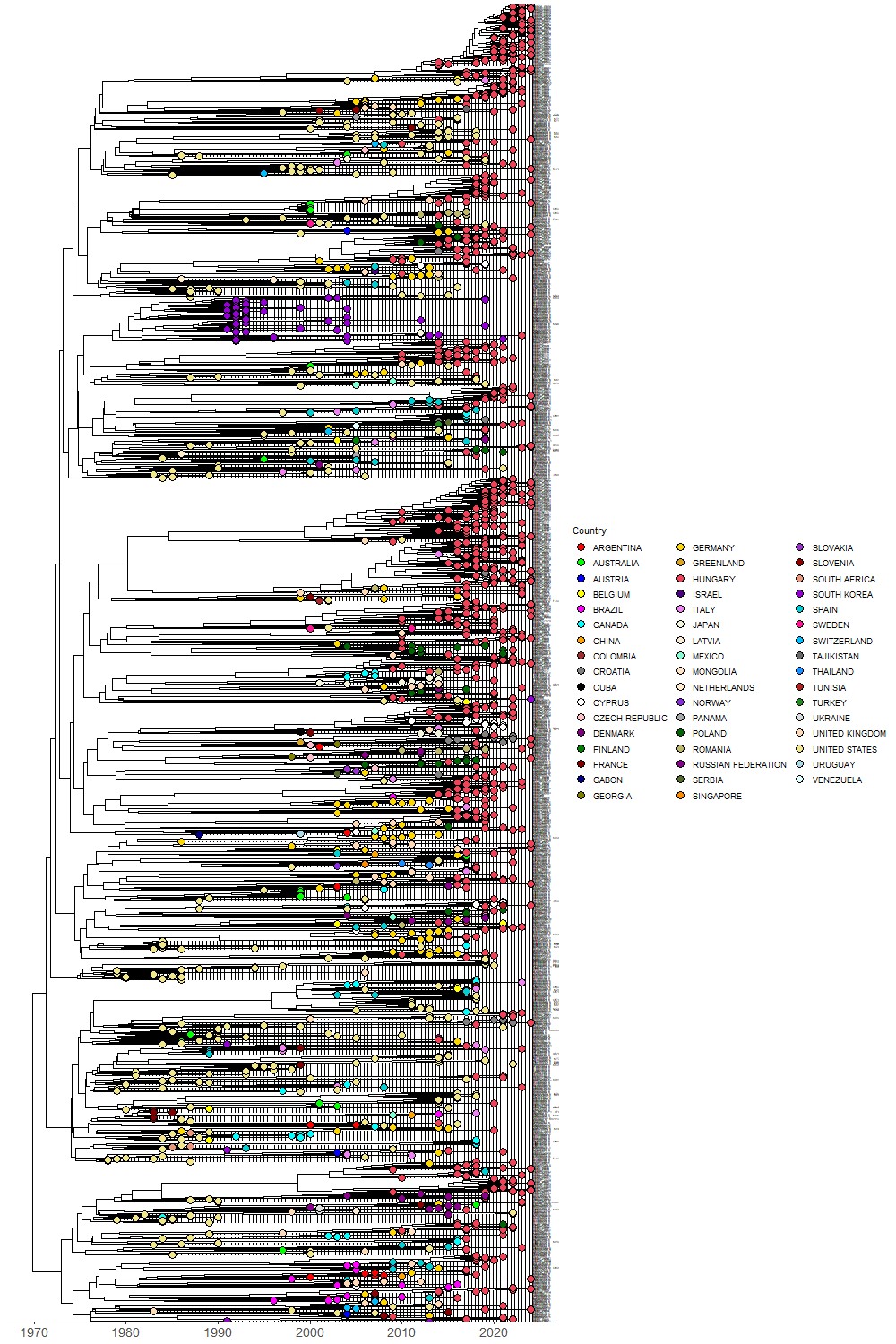

Supplement: Supplementary file 2 [file Data_Sheet_2.zip › HIVHUN_SFigure13300.jpg]

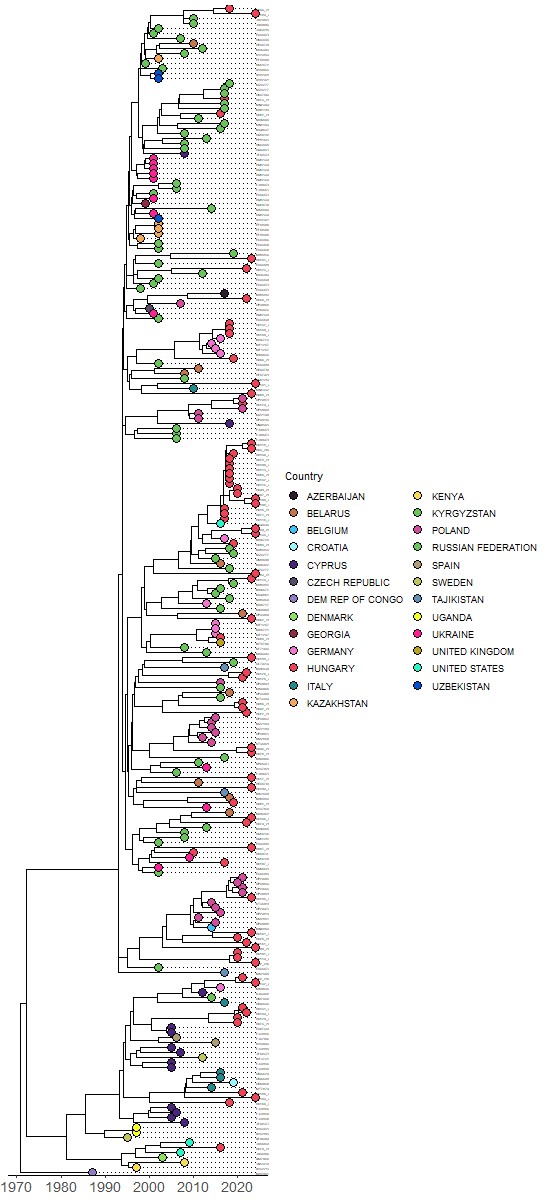

Supplement: Supplementary file 2 [file Data_Sheet_2.zip › HIVHUN_SFigure14300.jpg]

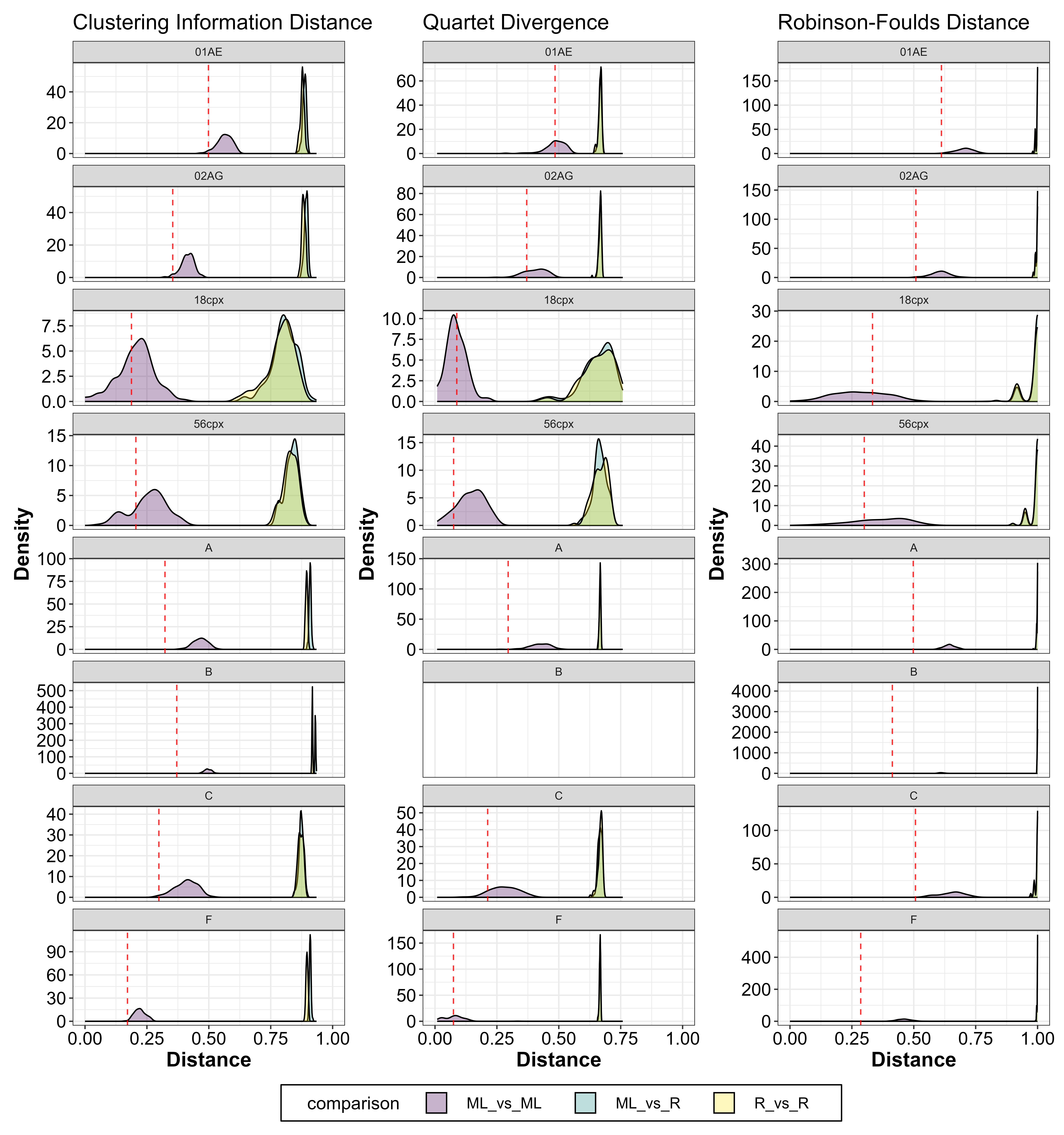

Supplement: Supplementary file 3 [file Data_Sheet_3.zip › HIVHUN_SFigure21300.jpg]

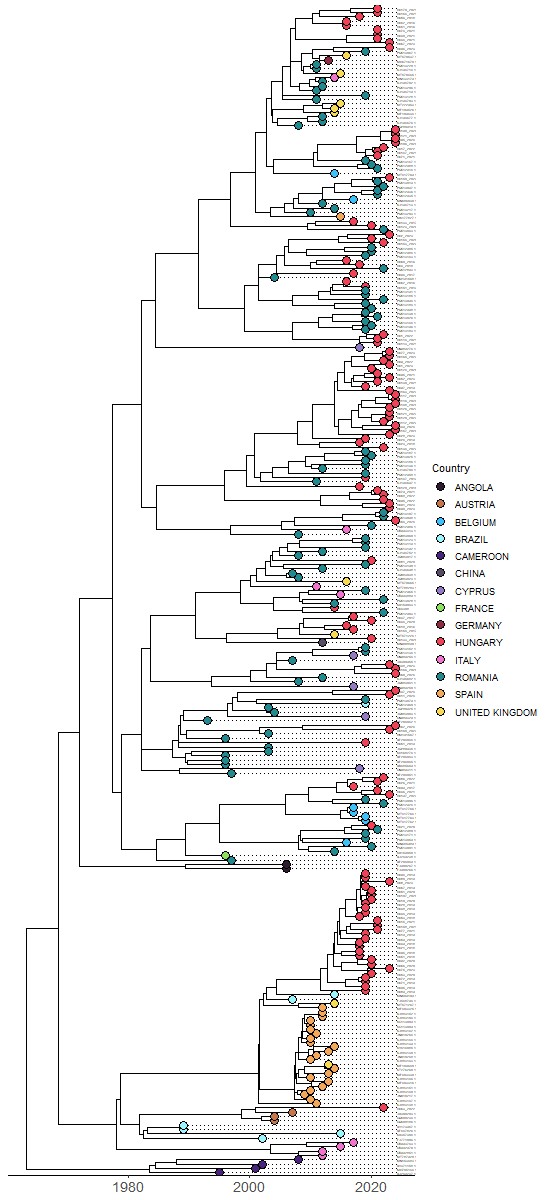

Supplement: Supplementary file 3 [file Data_Sheet_3.zip › HIVHUN_SFigure15300.jpg]

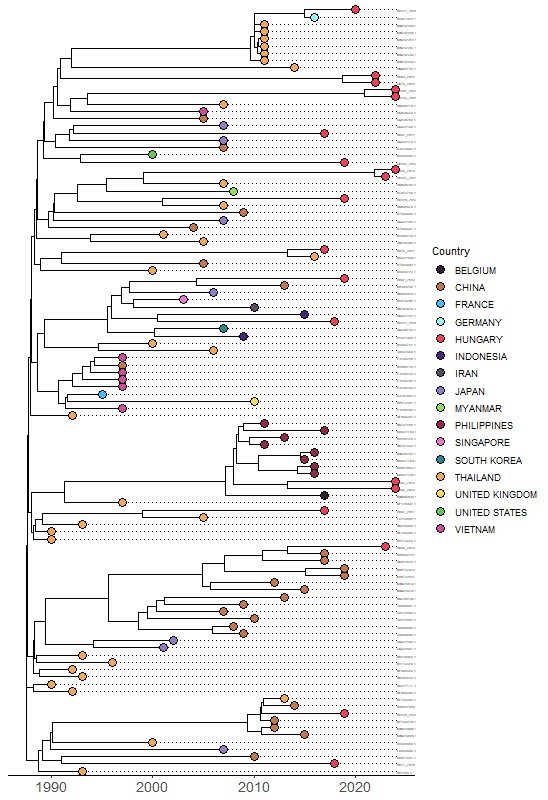

Supplement: Supplementary file 3 [file Data_Sheet_3.zip › HIVHUN_SFigure16300.jpg]

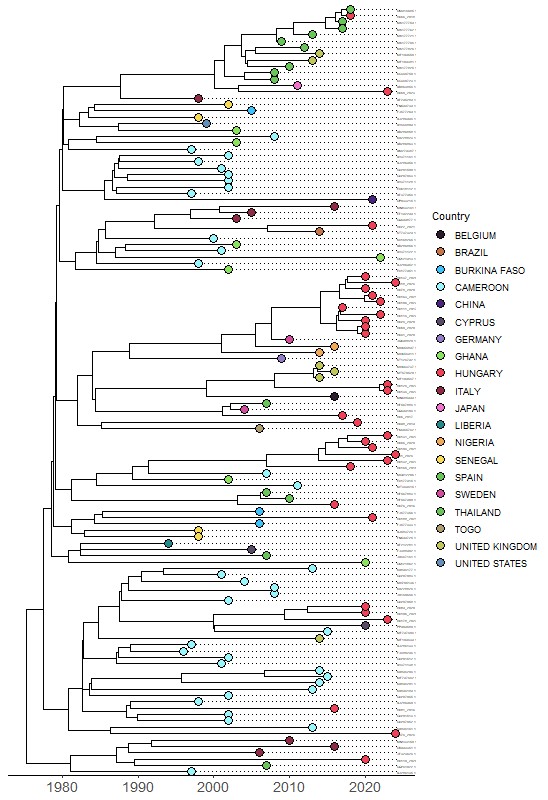

Supplement: Supplementary file 3 [file Data_Sheet_3.zip › HIVHUN_SFigure17300.jpg]

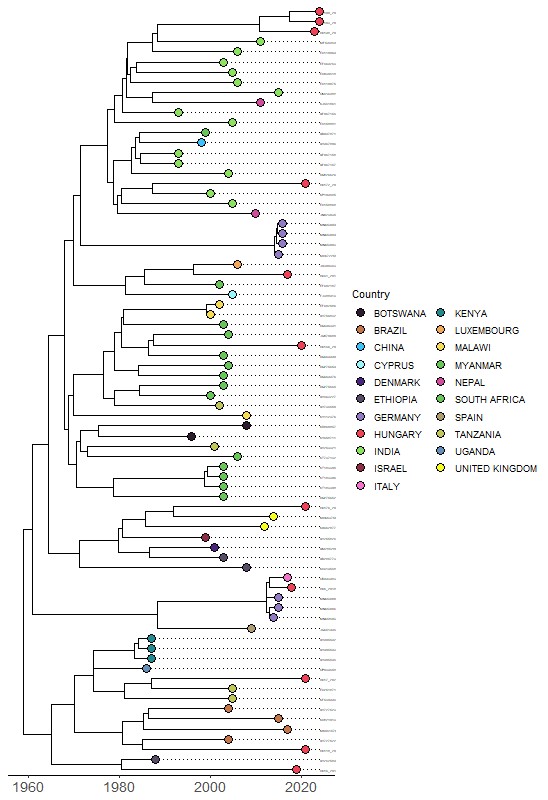

Supplement: Supplementary file 3 [file Data_Sheet_3.zip › HIVHUN_SFigure18300.jpg]

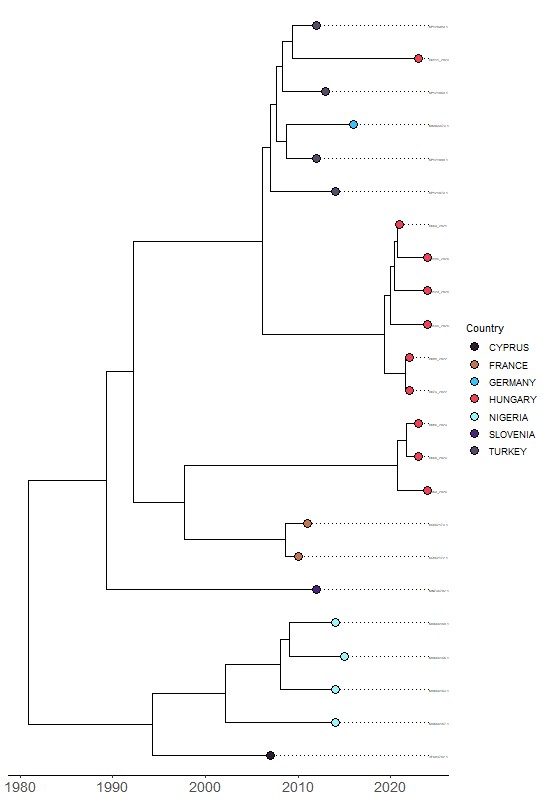

Supplement: Supplementary file 3 [file Data_Sheet_3.zip › HIVHUN_SFigure19300.jpg]

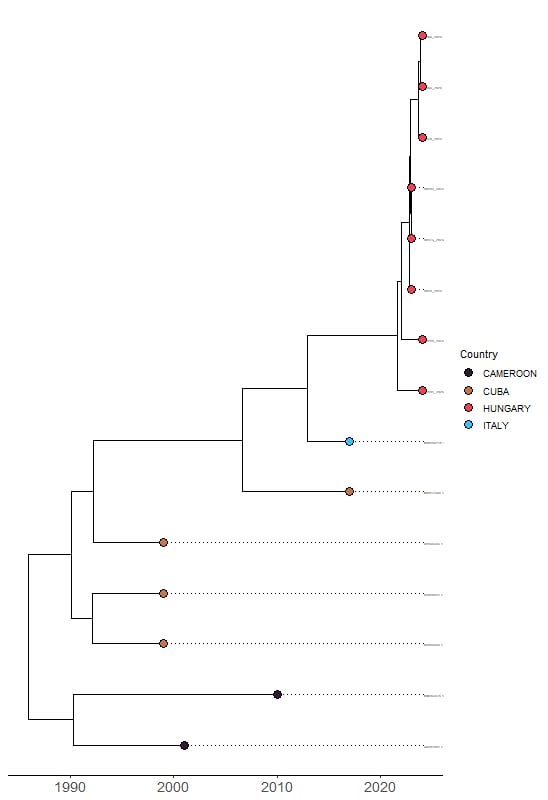

Supplement: Supplementary file 3 [file Data_Sheet_3.zip › HIVHUN_SFigure20300.jpg]
