# Supplementary material for: Molecular epidemiology of HIV-1 in Hungary: an evolving contact zone of colliding virus subtypes
Source: Front Microbiol. 2025 Dec 16;16:1732254. doi: 10.3389/fmicb.2025.1732254 (PMC12748157; doi:10.3389/fmicb.2025.1732254)
Supplement: Supplementary file 4 [file Data_Sheet_4.docx]

Supplementary Material

# Supplementary Tables

**Supplementary Table 1: Characteristics of the study population by cluster membership categories.** Abbreviations: MSM, men who have sex with men; HET, heterosexual contact; MTCT, maternal contact; PWID, people who inject drugs; UNK, unknown.

|  |  | Total population | | In large cluster (10+) | | In small cluster (2-9) | | Singleton | |
| --- | --- | --- | --- | --- | --- | --- | --- | --- | --- |
|  |  | n | % | n | % | n | % | n | % |
| Samples |  | 1120 | 100% | 442 | 39.5% | 404 | 36.1% | 274 | 24.5% |
| Sex | Male | 1034 | 92.3% | 436 | 98.6% | 374 | 92.6% | 224 | 81.8% |
|  | Female | 84 | 7.5% | 6 | 1.4% | 30 | 7.4% | 48 | 17.5% |
|  | UNK | 2 | 0.2% | 0 | 0% | 0 | 0% | 2 | 0.7% |
| Age group | **I** <20 | 20 | 1.8% | 8 | 1.8% | 8 | 2.0% | 4 | 1.5% |
|  | **II** 20-29 | 364 | 32.5% | 179 | 40.5% | 111 | 27.5% | 74 | 27.0% |
|  | **III** 30-39 | 372 | 33.2% | 143 | 32.4% | 140 | 34.7% | 89 | 32.5% |
|  | **IV** 40-49 | 207 | 18.5% | 71 | 16.1% | 85 | 21.0% | 51 | 18.6% |
|  | **V** >=50 | 109 | 9.7% | 31 | 7.0% | 41 | 10.1% | 37 | 13.5% |
|  | UNK | 48 | 4.3% | 10 | 2.3% | 19 | 4.7% | 19 | 6.9% |
| Route of infection | MSM | 951 | 84.9% | 413 | 93.4% | 340 | 84.2% | 198 | 72.3% |
|  | HET | 112 | 10.0% | 13 | 2.9% | 40 | 9.9% | 59 | 21.5% |
|  | MTCT | 7 | 0.6% | 0 | 0% | 5 | 1.2% | 2 | 0.7% |
|  | PWID | 1 | 0.1% | 0 | 0% | 0 | 0% | 1 | 0.4% |
|  | UNK | 49 | 4.4% | 16 | 3.6% | 19 | 4.7% | 14 | 5.1% |
| CD4+ T cell count (cells/mm^3^) | <200 | 212 | 18.9% | 41 | 9.3% | 88 | 21.8% | 83 | 30.3% |
|  | >=200 | 824 | 73.6% | 382 | 86.4% | 285 | 70.5% | 157 | 57.3% |
|  | UNK | 84 | 7.5% | 19 | 4.3% | 31 | 7.7% | 34 | 12.4% |
| Subtype | B | 815 | 72.8% | 355 | 80.3% | 284 | 70.3% | 176 | 64.2% |
|  | F | 112 | 10.0% | 48 | 10.9% | 48 | 11.9% | 16 | 5.8% |
|  | A | 71 | 6.3% | 16 | 3.6% | 25 | 6.2% | 30 | 10.9% |
|  | CRF02_AG | 31 | 2.8% | 10 | 2.3% | 7 | 1.7% | 14 | 5.1% |
|  | CRF01_AE | 19 | 1.7% | 0 | 0% | 8 | 2.0% | 11 | 4.0% |
|  | CRF18_cpx | 12 | 1.1% | 12 | 2.7% | 0 | 0% | 0 | 0% |
|  | C | 11 | 1.0% | 0 | 0% | 2 | 0.5% | 9 | 3.3% |
|  | G | 2 | 0.2% | 0 | 0% | 0 | 0% | 2 | 0.7% |
|  | CRF06_cpx | 2 | 0.2% | 0 | 0% | 2 | 0.5% | 0 | 0% |
|  | CRF19_cpx | 2 | 0.2% | 0 | 0% | 2 | 0.5% | 0 | 0% |
|  | non-typable | 43 | 3.8% | 1 | 0.2% | 26 | 6.4% | 16 | 5.8% |

# Supplementary Figures


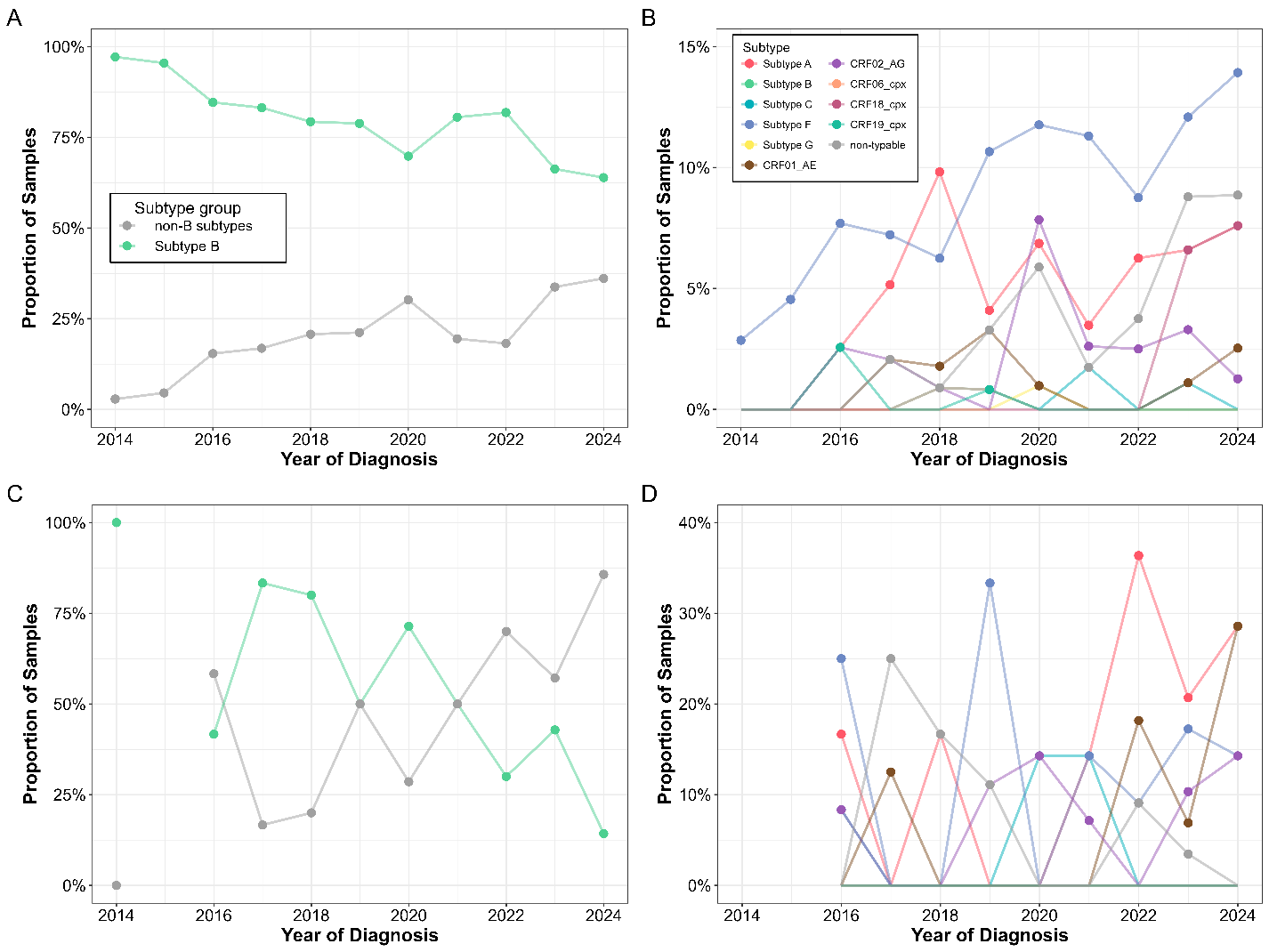


**Supplementary Figure 1: Subtyping results for MSM and HET risk groups in Hungary until 2024.** (A) The ratio of subtype B and non-B subtypes among MSM sequences from Hungary between 2014-2024. (B) Distribution of non-B subtype sequences in the MSM risk group by year between 2014 and 2024. (C) The ratio of subtype B and non-B subtypes among HET sequences from Hungary between 2014 and 2024. (D) Distribution of non-B subtype sequences in the HET risk group by year between 2014 and 2024.


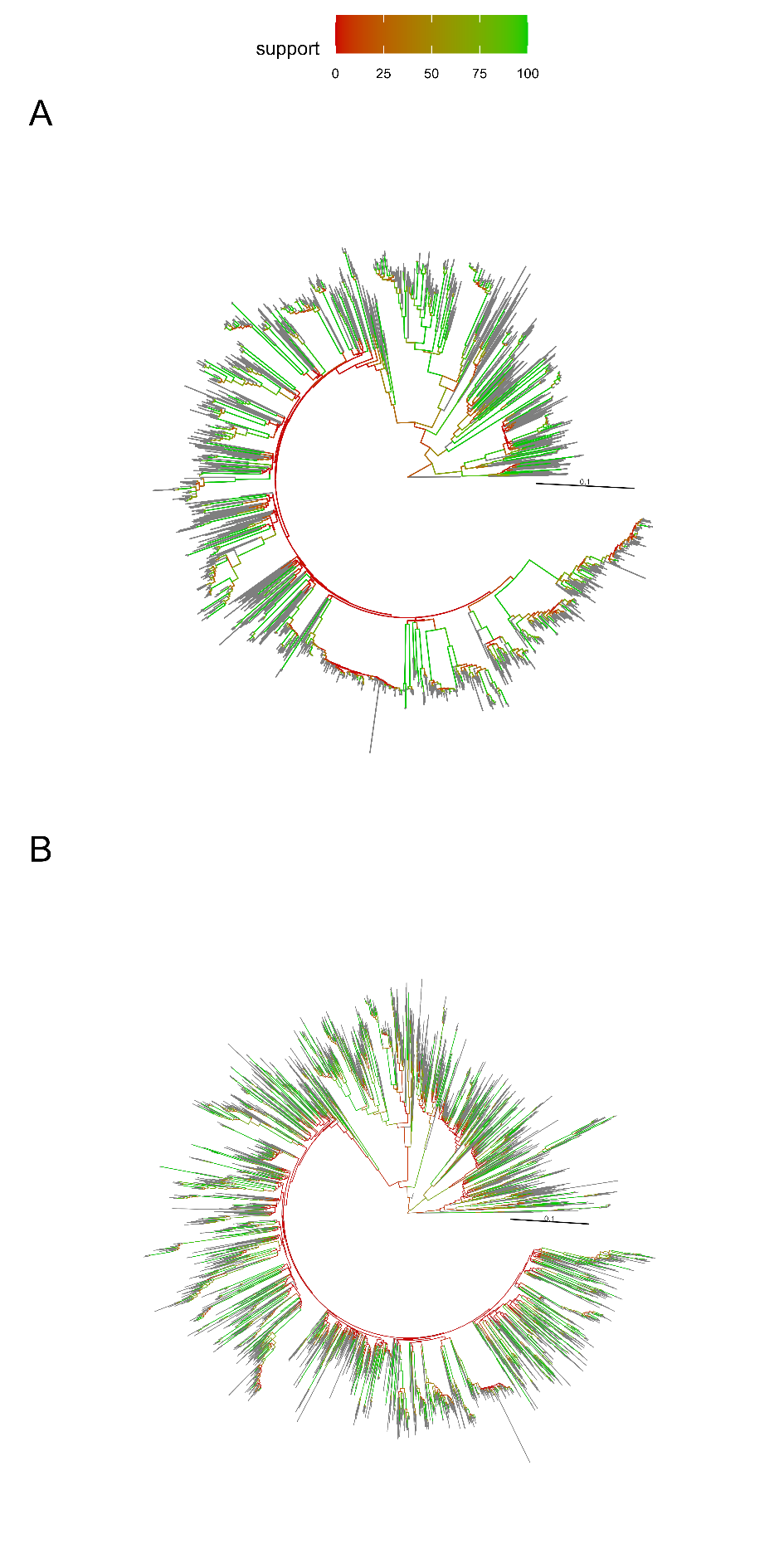
Supplementary Figure 2: Branch support for the constructed maximum likelihood phylogenetic trees. **(A)** A maximum likelihood phylogenetic tree built from HIV-1 sequences obtained from Hungarian HIV-positive individuals up to the end of 2024 and a reference dataset encompassing subtypes and CRFs present in Hungary, and **(B)** a tree constructed from Hungarian, reference, and international background sequences queried from the NCBI Nucleotide database. Edges are colored according to their branch support.


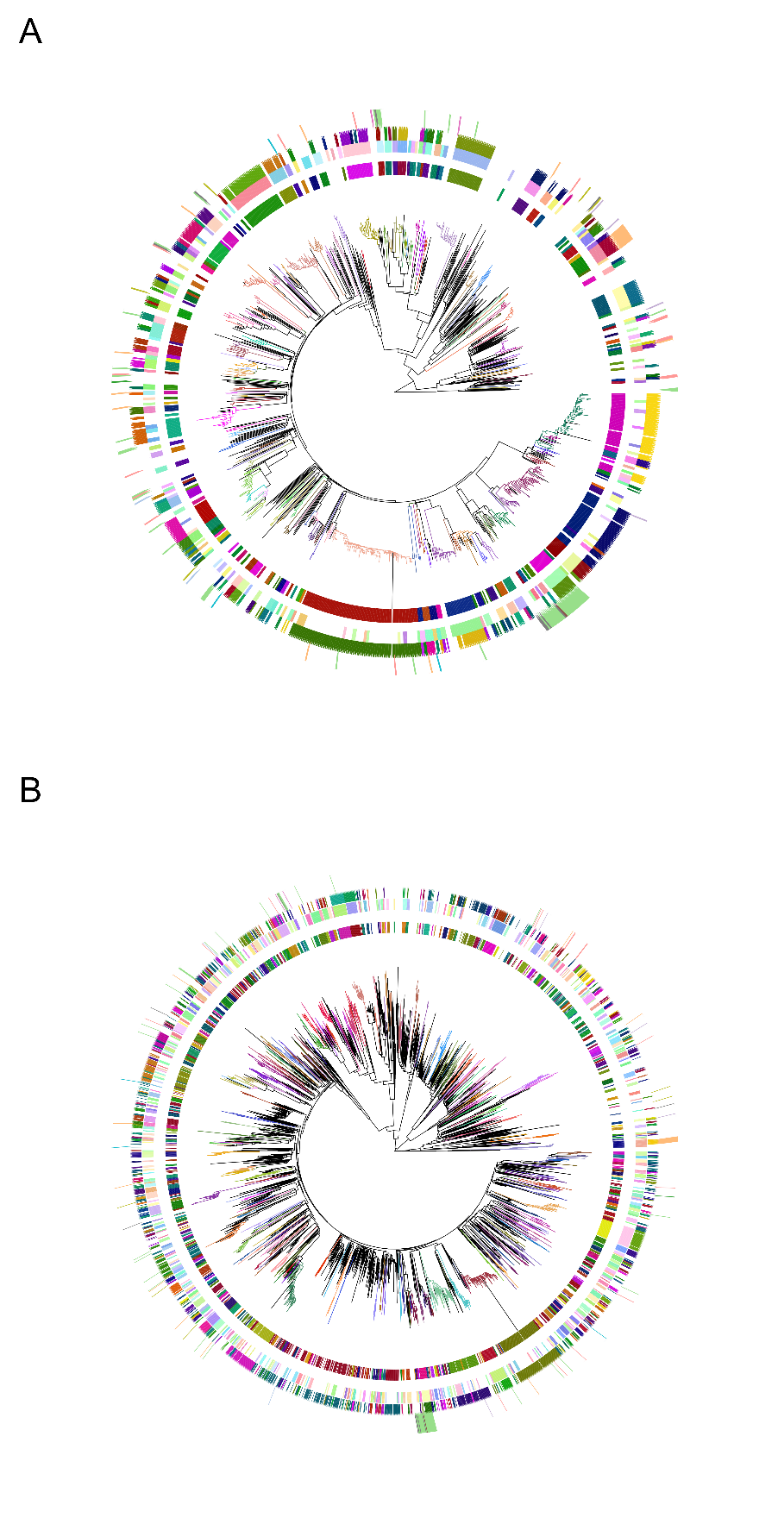
Supplementary Figure 3: Comparison of clustering annotations for the constructed maximum likelihood phylogenetic trees. **(A)** A maximum likelihood phylogenetic tree built from HIV-1 sequences obtained from Hungarian HIV-positive individuals up to the end of 2024, along with a reference dataset encompassing subtypes and CRFs present in Hungary, and **(B)** a tree constructed from Hungarian sequences, reference sequences, and additional international background sequences queried from the NCBI Nucleotide database. Branches are colored according to transmission clusters identified using a combination of ClusterPicker (maximum genetic distance of 0.045, branch support threshold of 0.80) and HIV-TRACE (pairwise distance threshold of 0.01). Colored concentric circles indicate the following annotations for each sequence (from innermost to outermost): (1) clusters identified using the combination of ClusterPicker and HIV-TRACE, (2) clusters identified by ClusterPicker (maximum genetic distance of 0.045, branch support threshold of 0.80), (3) clusters identified by HIV-TRACE (pairwise distance threshold of 0.01), and (4) surveillance drug resistance mutations grouped by drug class (PI = protease inhibitor, NRTI = nucleoside reverse transcriptase inhibitor, NNRTI = non-nucleoside reverse transcriptase inhibitor, INI = integrase inhibitor).


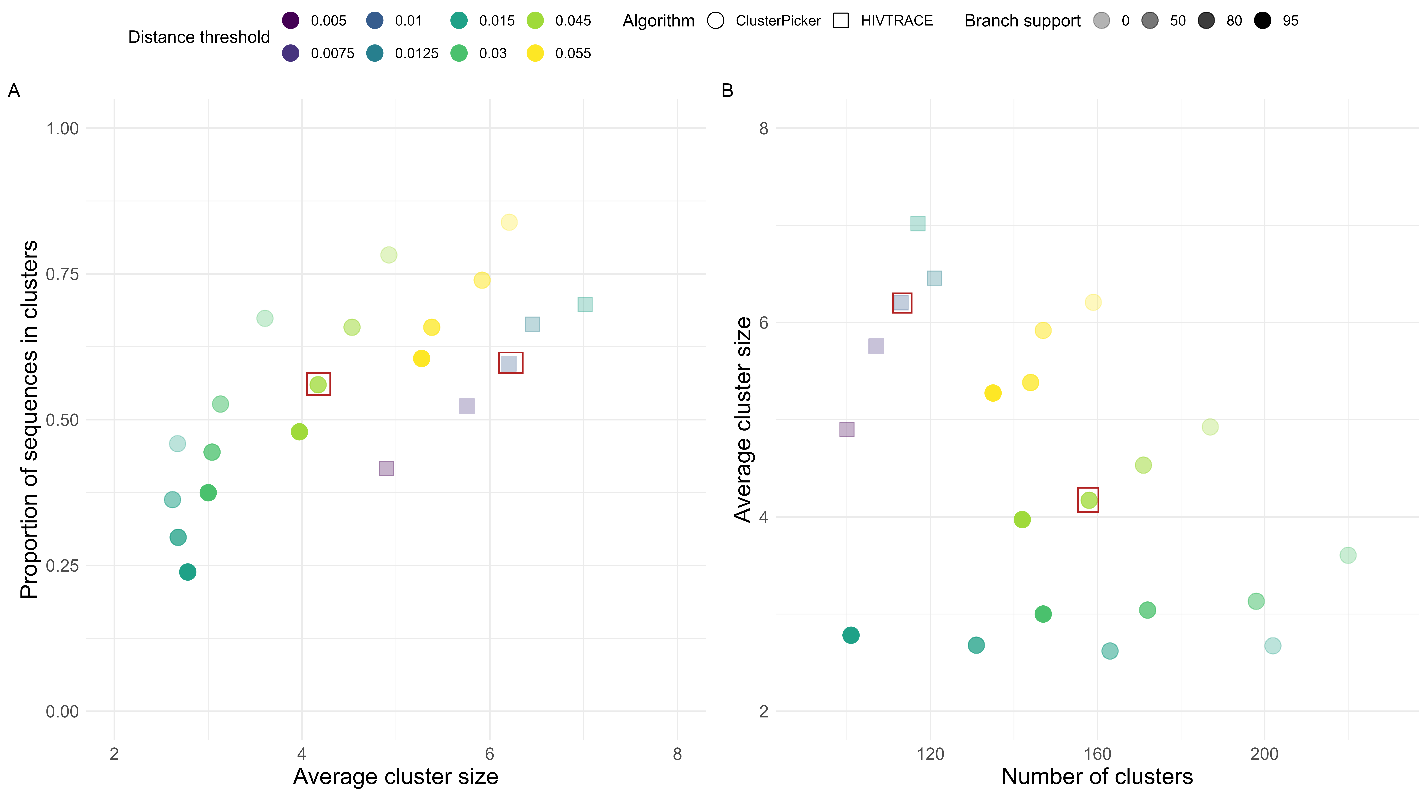


Supplementary Figure 4: Impact of clustering algorithms, genetic distance, and branch support thresholds on cluster characteristics generated from the maximum likelihood phylogenetic tree of Hungarian HIV-1 sequences. **(A)** Relationship between the proportion of sequences in clusters and the average cluster size. **(B)** Relationship between average cluster size and the total number of clusters. Different algorithms are represented by distinct point shapes, while variations in genetic distance thresholds and branch support thresholds are indicated by color and opacity, respectively. Parameter combinations for ClusterPicker and HIV-TRACE used to generate the primary results are highlighted with red rectangles.


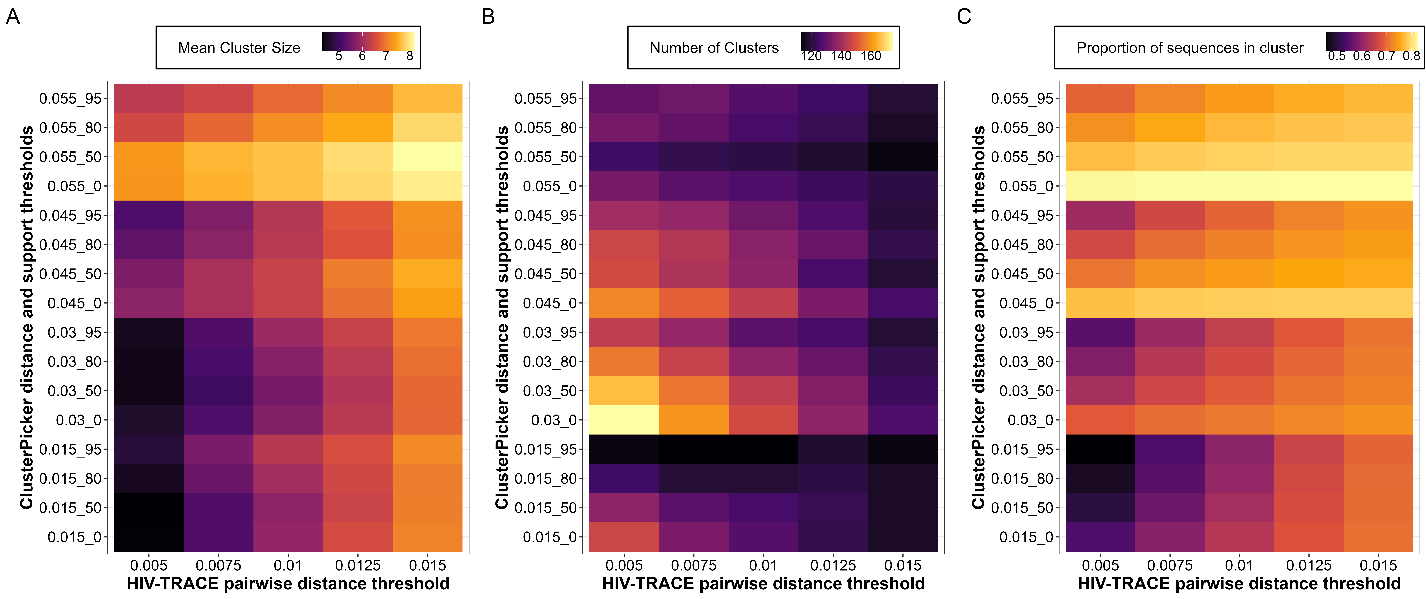


Supplementary Figure 5: Impact of genetic distance and branch support thresholds on cluster characteristics generated using the combination of ClusterPicker and HIV-TRACE clustering tools from the maximum likelihood phylogenetic tree of Hungarian HIV-1 sequences. **(A)** Mean cluster size, **(B)** total number of clusters, and **(C)** proportion of sequences in clusters across different parameter combinations of ClusterPicker (genetic distance and branch support thresholds) and HIV-TRACE (pairwise genetic distance threshold).


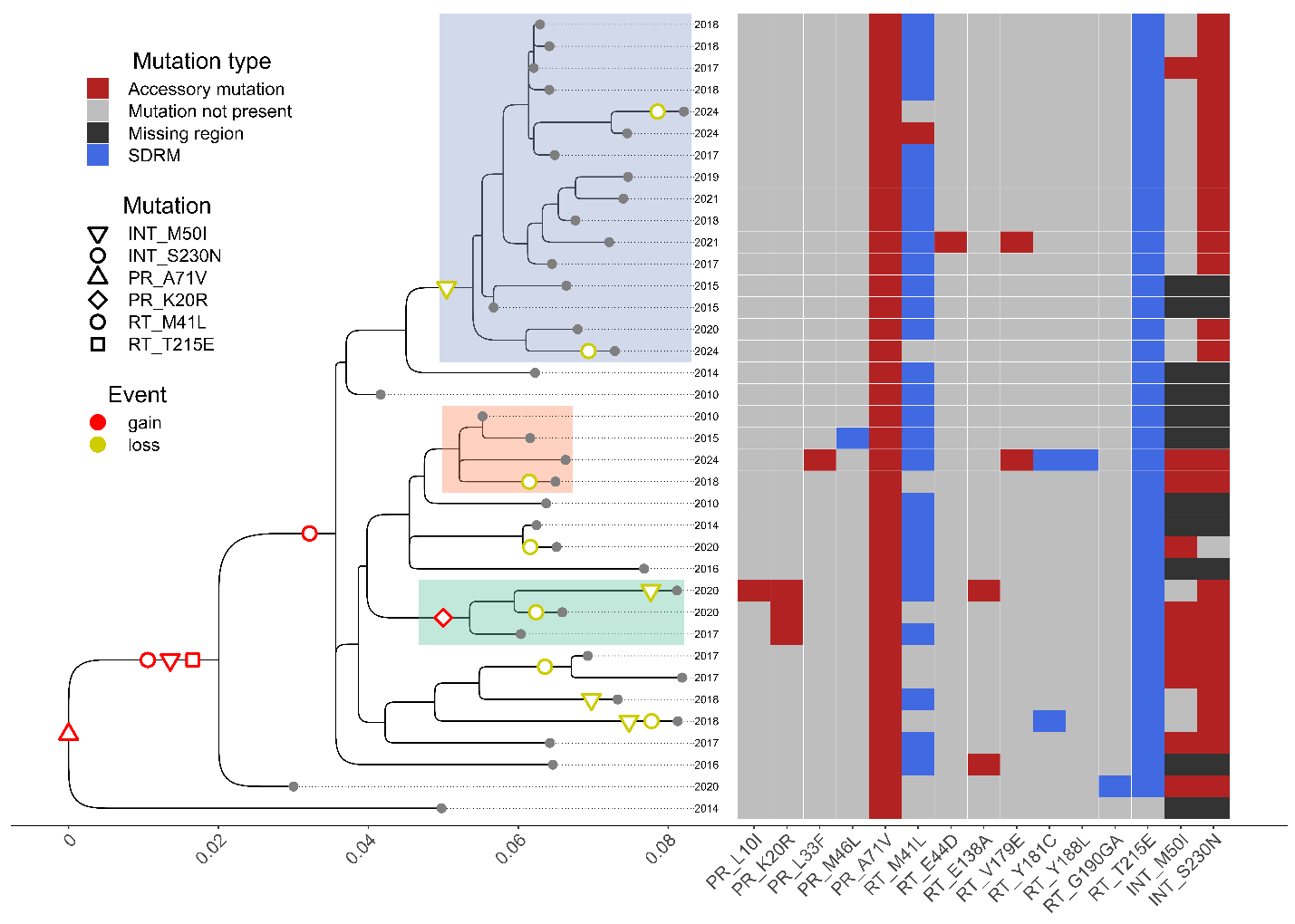
Supplementary Figure 6. Maximum-likelihood phylogenetic tree of a long-lived TDR cluster in Hungary. Transmission clusters identified by our molecular clustering approach are shaded in blue, orange, and green on the phylogeny. The presence or absence of observed SDRMs and polymorphisms is displayed as a tile plot for each sequence in the cluster. The gain (red border) and loss (yellow border) of specific mutations along the phylogeny were inferred using a maximum parsimony approach and are indicated by distinct symbols for each mutation.


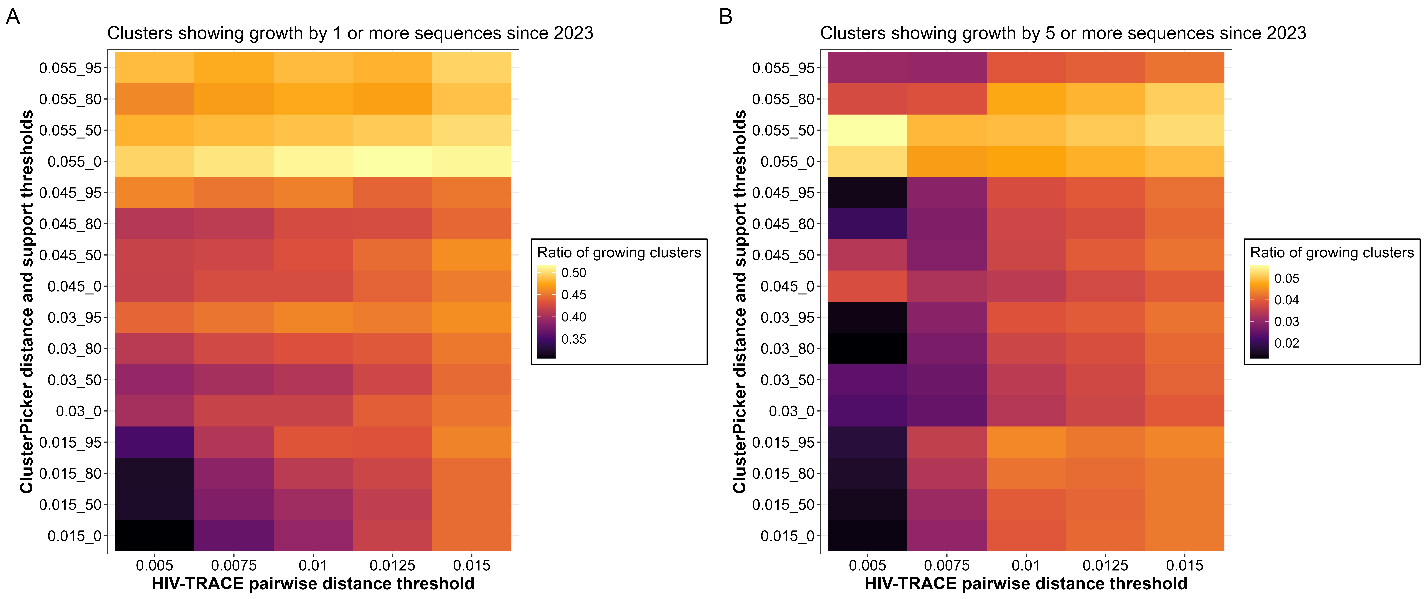
Supplementary Figure 7: Impact of genetic distance and branch support thresholds on cluster growth using the combination of ClusterPicker and HIV-TRACE clustering tools from the maximum likelihood phylogenetic tree of Hungarian HIV-1 sequences. **(A)** Ratio of clusters growing since 2023 by at least one sequence **(B)** or 5 sequences. Cluster growth is presented across different parameter combinations of ClusterPicker (genetic distance and branch support thresholds) and HIV-TRACE (pairwise genetic distance threshold).


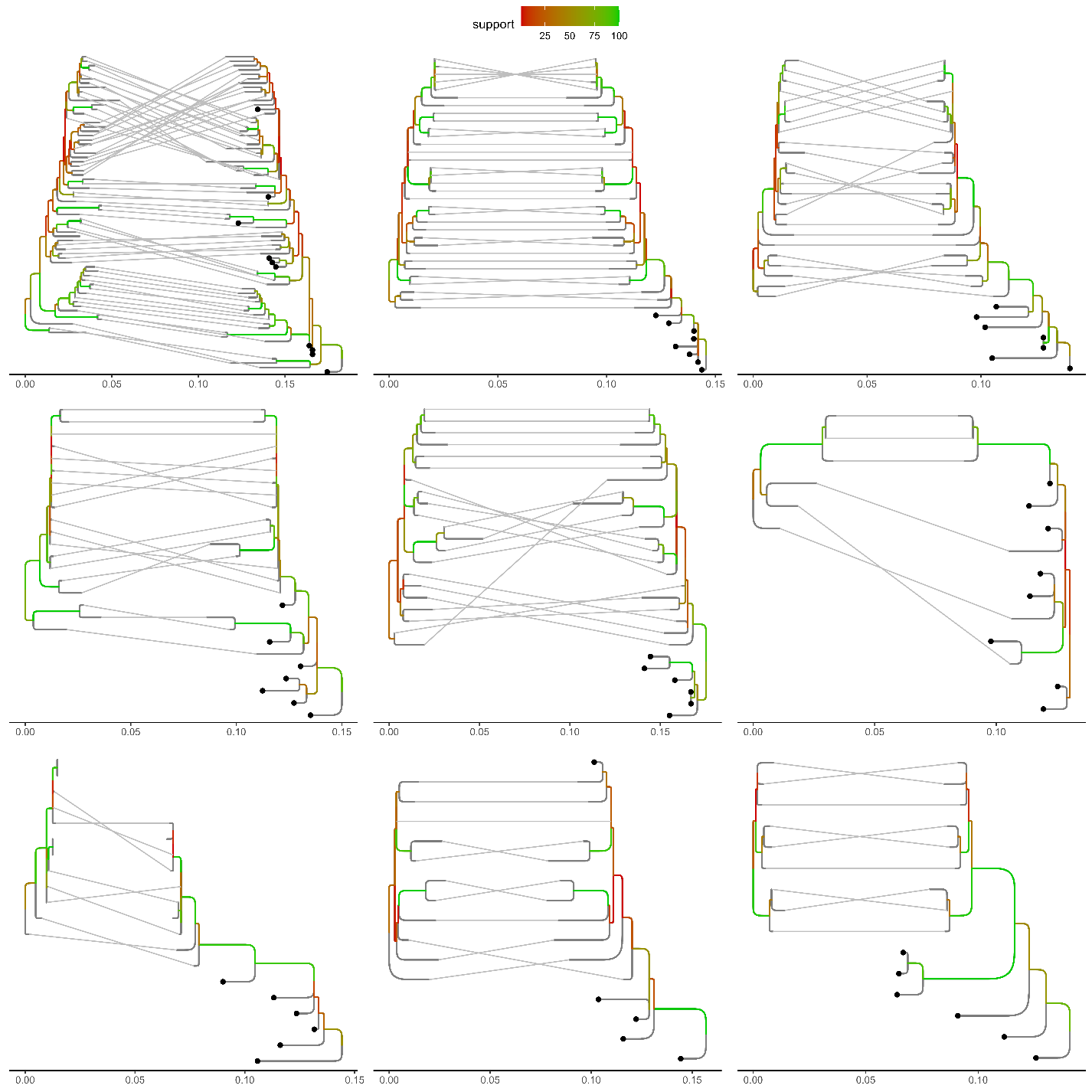
Supplementary Figure 8: Comparison of HIV-1 sequence clusters between phylogenetic trees constructed from Hungarian sequences alone (left tree) and those including international background sequences (right tree). Clusters depicted meet the criteria of having a size greater than 15 and containing at least 25% Hungarian sequences. Background sequences are marked with black circles at the tips. Corresponding Hungarian sequences in both trees are connected by gray lines. Branches are colored according to their phylogenetic support.


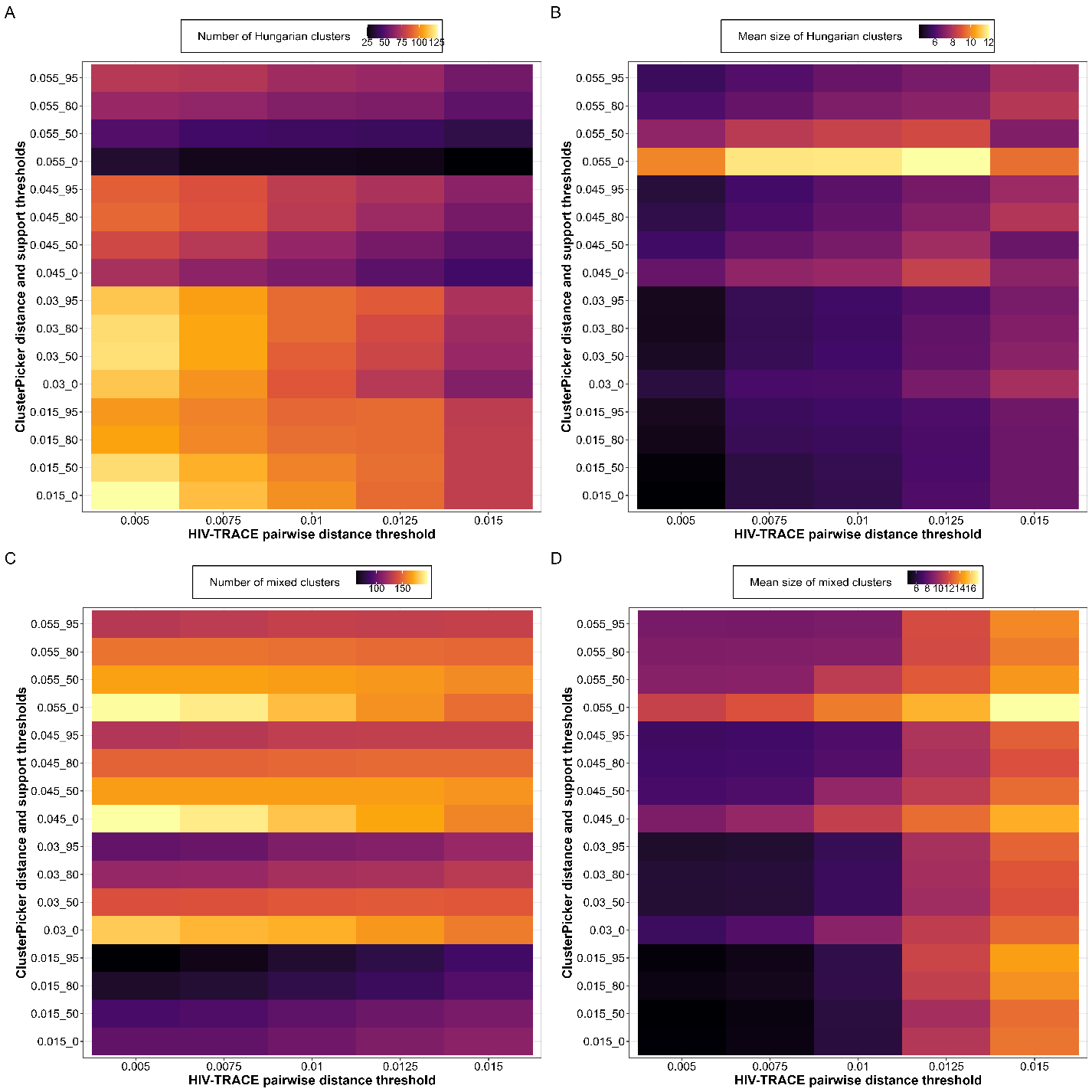


Supplementary Figure 9: Impact of genetic distance and branch support thresholds on cluster characteristics generated using the combination of ClusterPicker and HIV-TRACE clustering tools from the maximum likelihood phylogenetic tree of Hungarian and international background HIV-1 sequences. **(A)** Number of clusters and **(B)** mean cluster size for Hungarian sequence clusters. **(C)** Number of clusters and **(D)** mean cluster size for mixed clusters containing at least one Hungarian and one international sequence. Metrics are presented across different parameter combinations of ClusterPicker (genetic distance and branch support thresholds) and HIV-TRACE (pairwise genetic distance threshold).


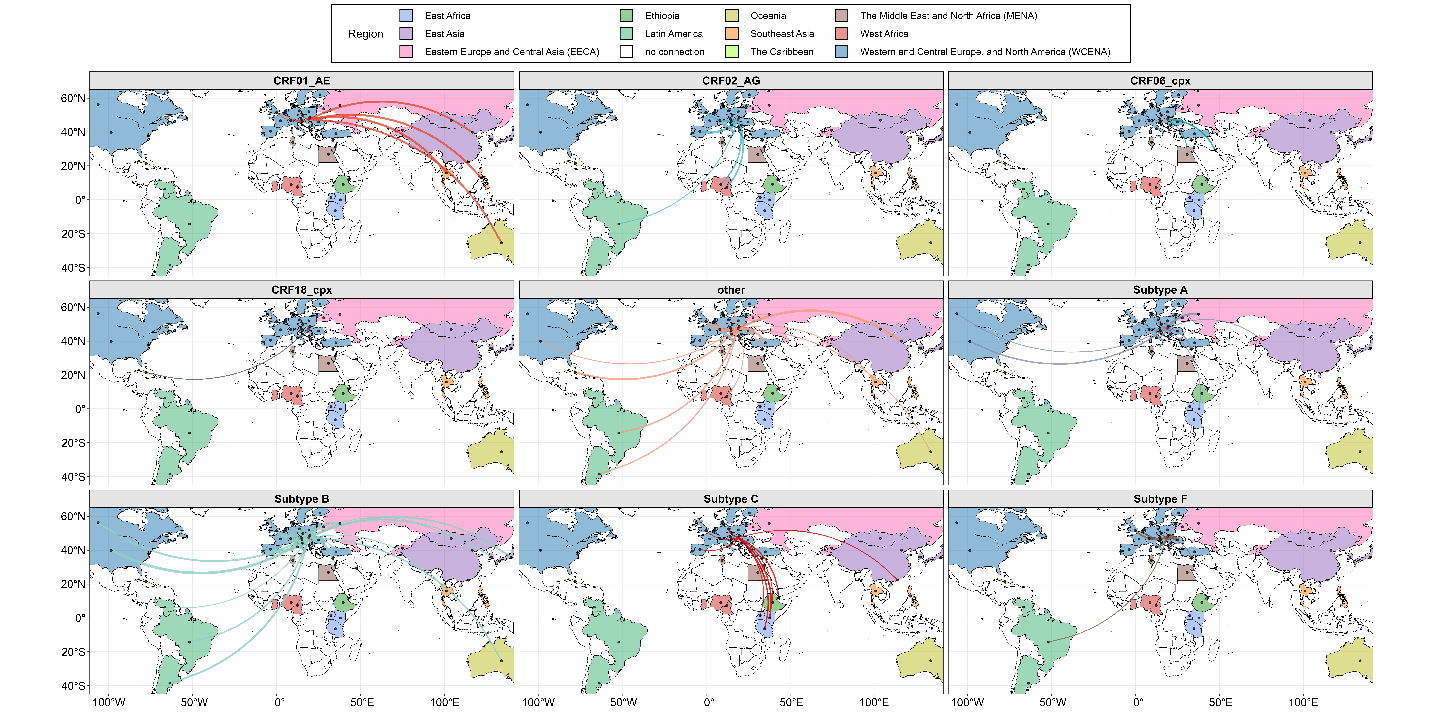
Supplementary Figure 10: Cross-country connections of Hungarian HIV-1 sequences based on mixed sequence clusters by subtype of sequence. Mixed clusters (containing at least one Hungarian and one international sequence) were identified using a combination of ClusterPicker (maximum genetic distance of 0.045 and branch support threshold of 0.80) and HIVTRACE (pairwise distance threshold of 0.01) clustering tools. Cross-border connections of Hungarian sequences within mixed clusters are displayed worldwide and colored by geographical region. The width of the lines is proportional to the number of international sequences in mixed clusters per country, while point sizes correspond to the number of sequences uploaded to the Los Alamos HIV Database by each country (accessed on February 10, 2025).


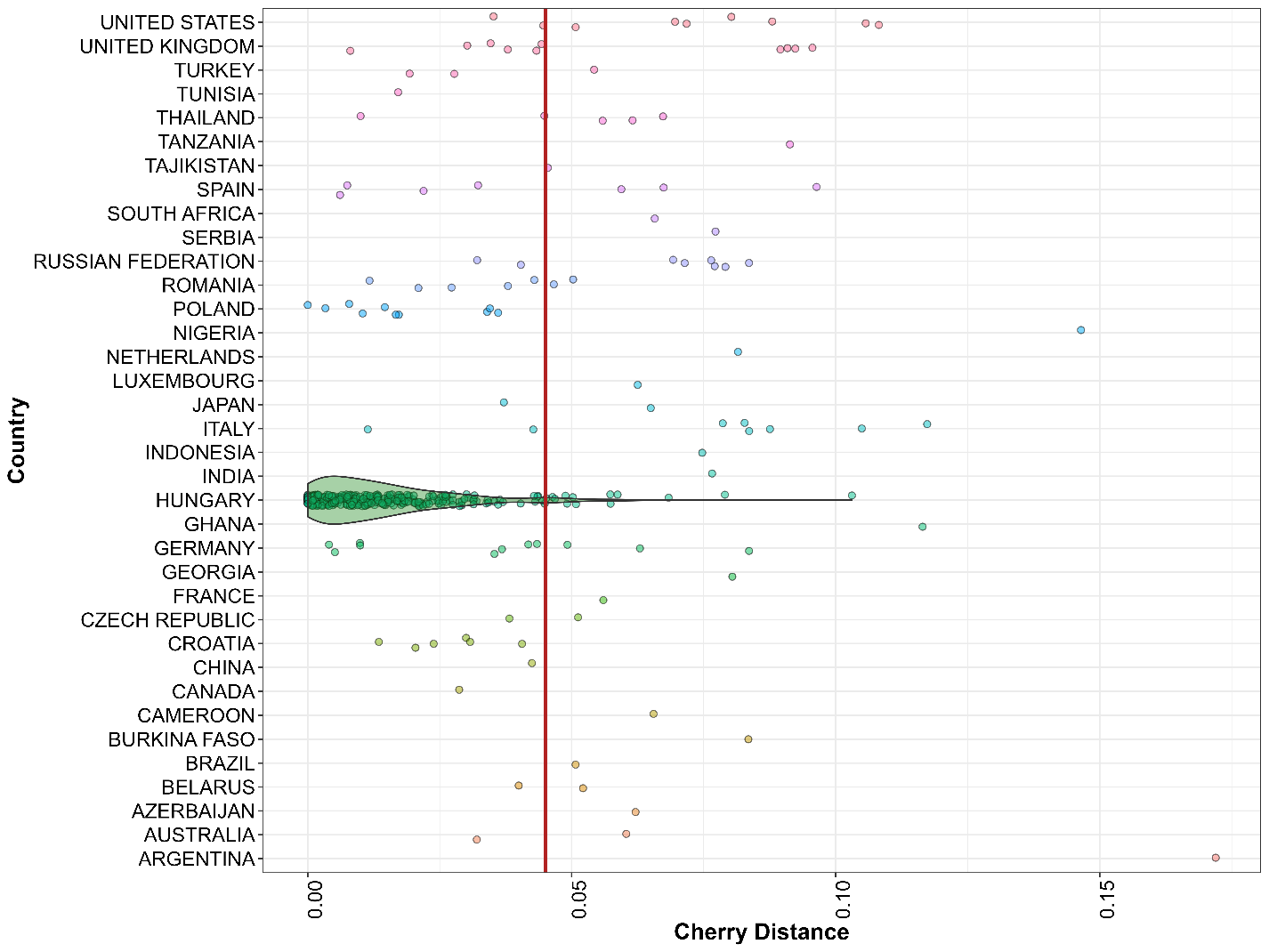
Supplementary Figure 11: Genetic distance and country of origin of identified Hungarian and mixed cherries. The cophenetic distance threshold of 0.045 used in the main analysis to define cherries is indicated by a red vertical line. The distribution of genetic distance values for Hungarian cherries is represented using a violin plot


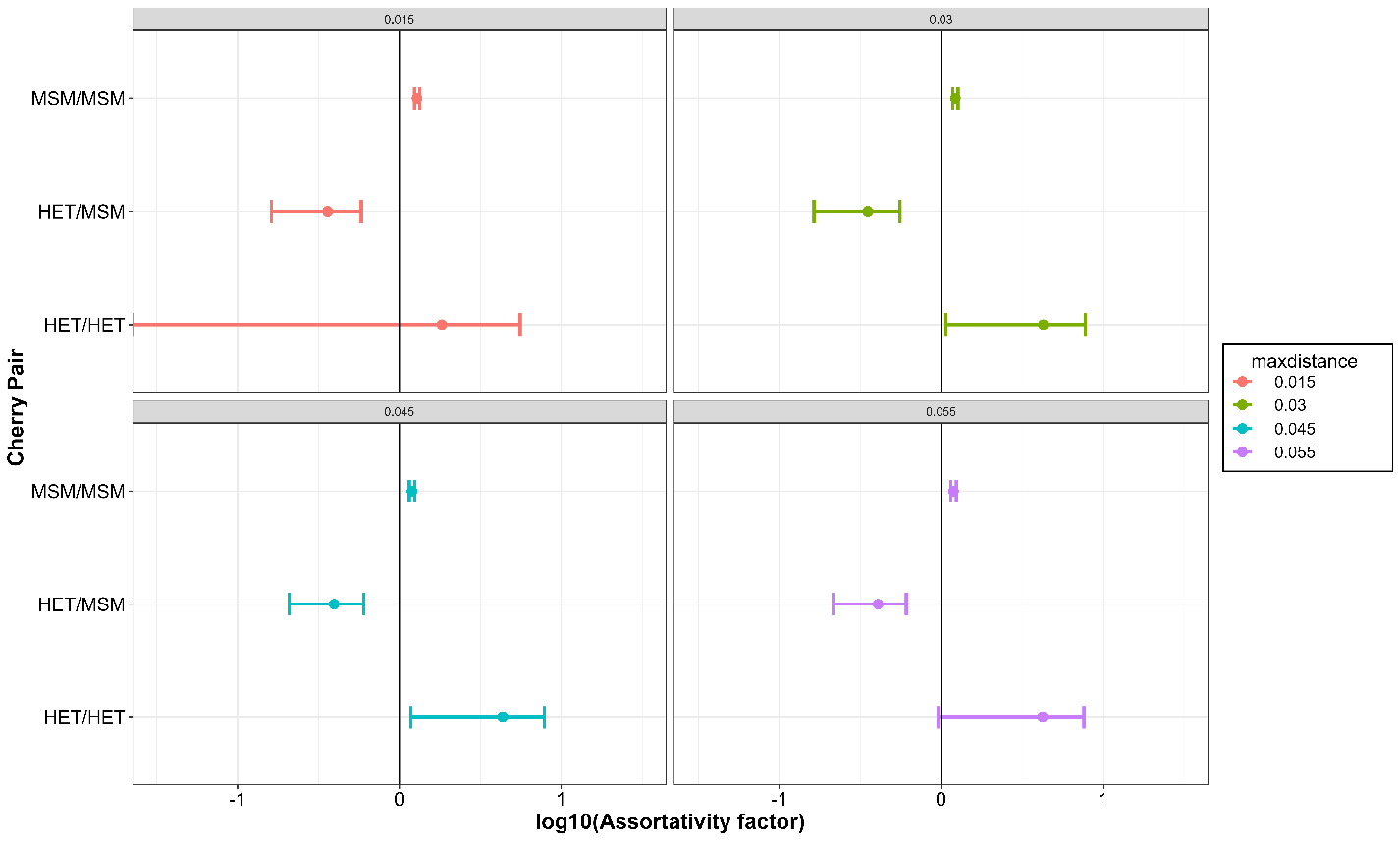
Supplementary Figure 12: Effect of genetic distance thresholds on risk group assortativity in Hungarian cherries. The assortativity factor (AF) above 1 indicates a higher occurrence of cherry pairs with a given risk group combination than expected under random pairing of tips, while AF < 1 indicates overdispersion compared to random pairing. Risk group abbreviations: MSM = men who have sex with men and HET = heterosexual. Error bars indicate 95% confidence intervals.


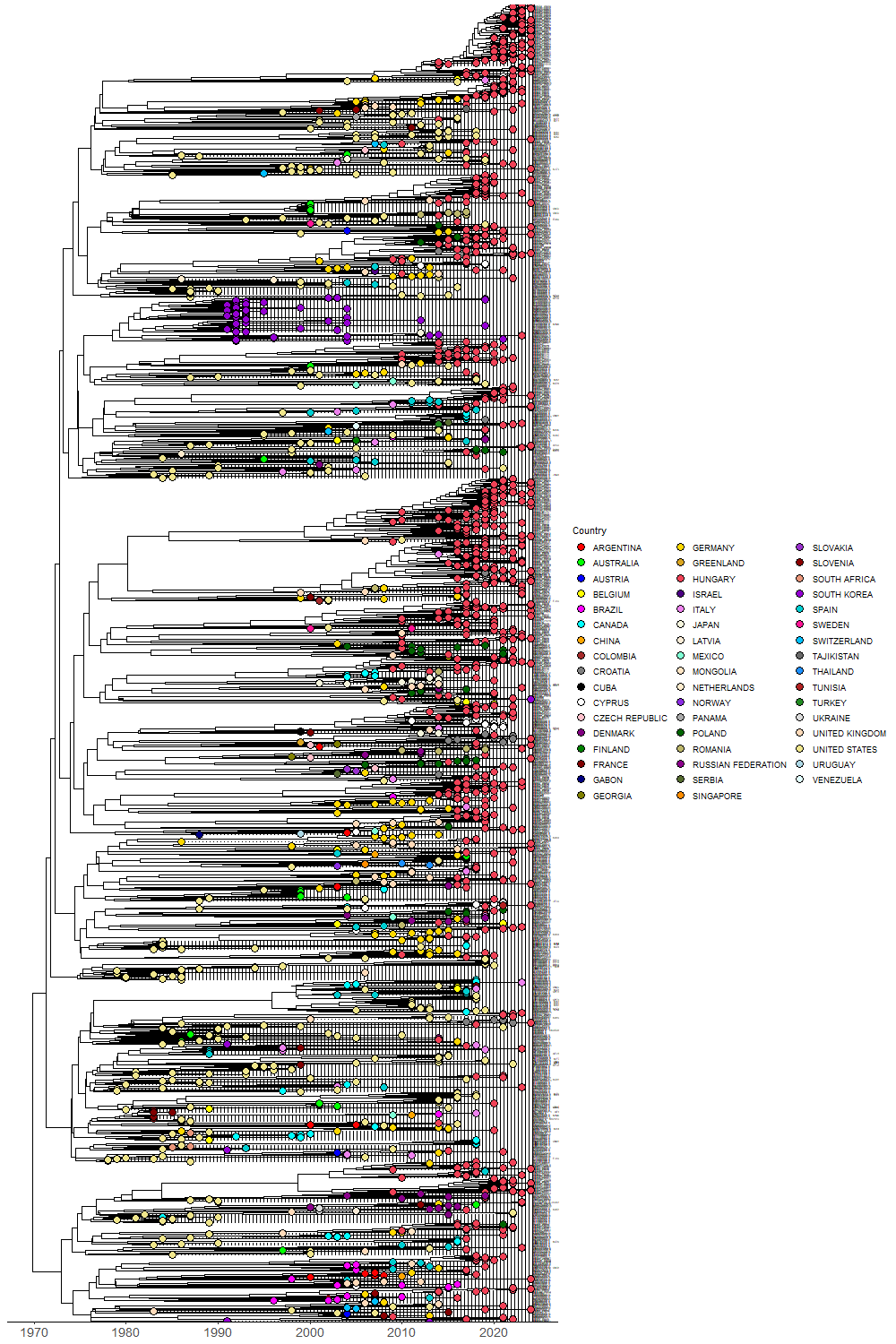
Supplementary Figure 13: Bayesian MCC tree from Hungarian and international HIV-1 sequences for subtype B. Tips are colored according to country of origin.


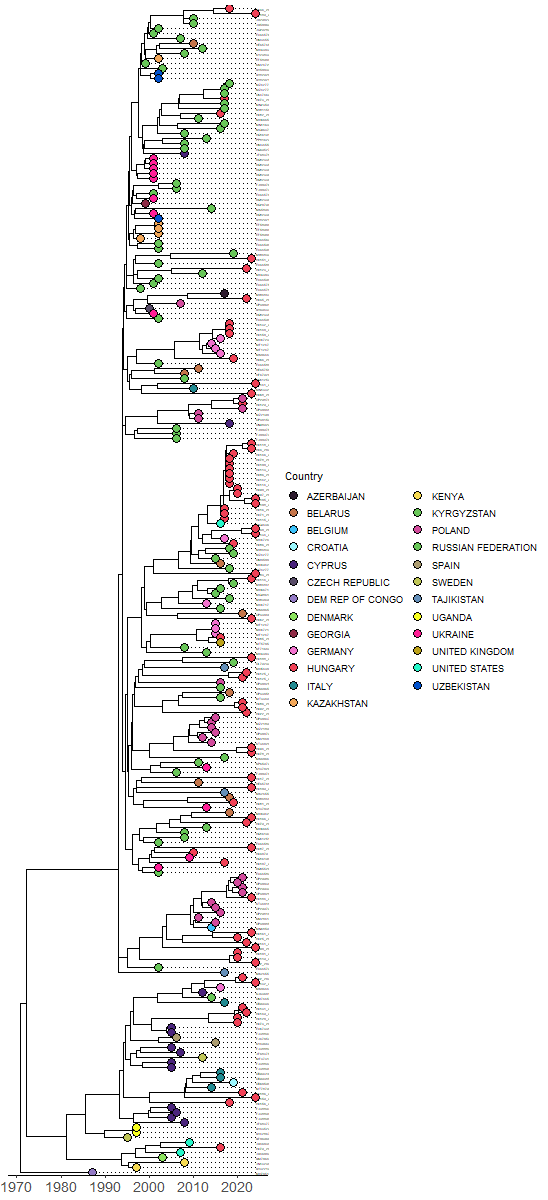
Supplementary Figure 14: Bayesian MCC tree from Hungarian and international HIV-1 sequences for subtype A. Tips are colored according to country of origin.


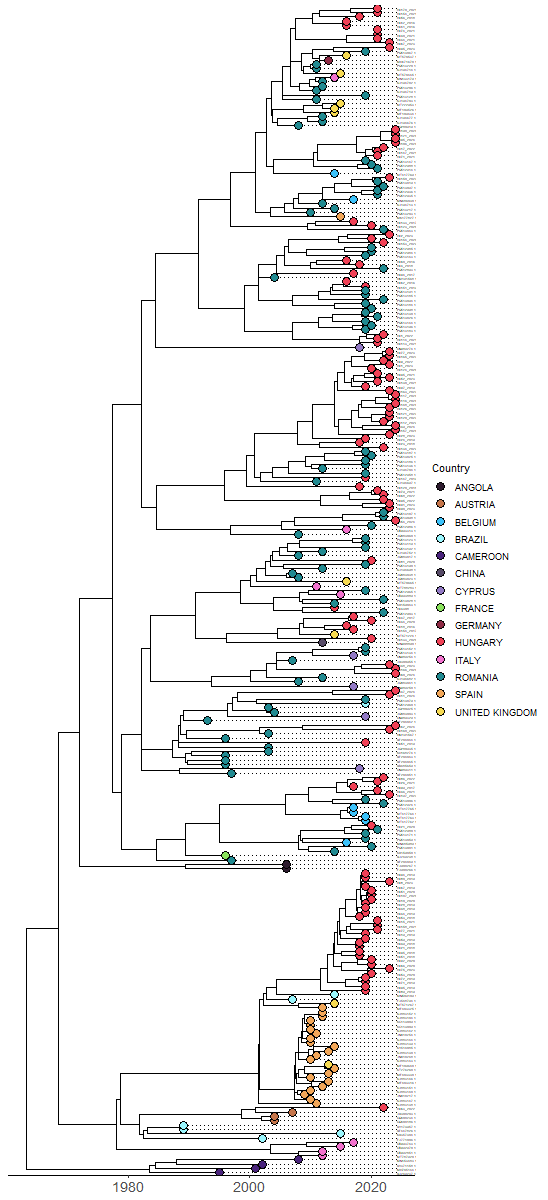
Supplementary Figure 15: Bayesian MCC tree from Hungarian and international HIV-1 sequences for subtype F. Tips are colored according to country of origin.


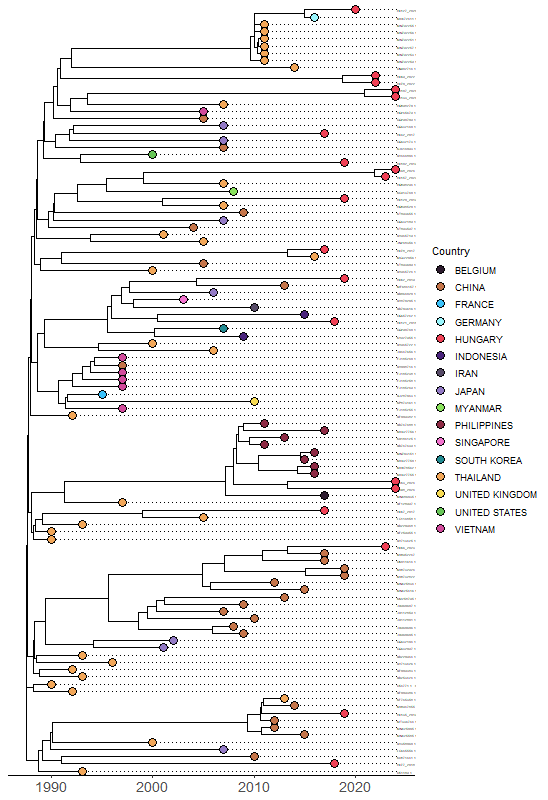
Supplementary Figure 16: Bayesian MCC tree from Hungarian and international HIV-1 sequences for CRF01_AE. Tips are colored according to country of origin.


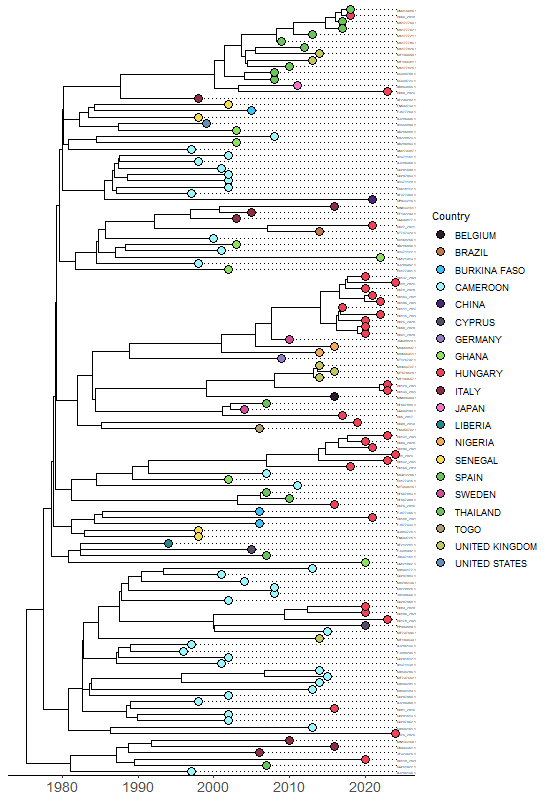
Supplementary Figure 17: Bayesian MCC tree from Hungarian and international HIV-1 sequences for CRF02_AG. Tips are colored according to country of origin.


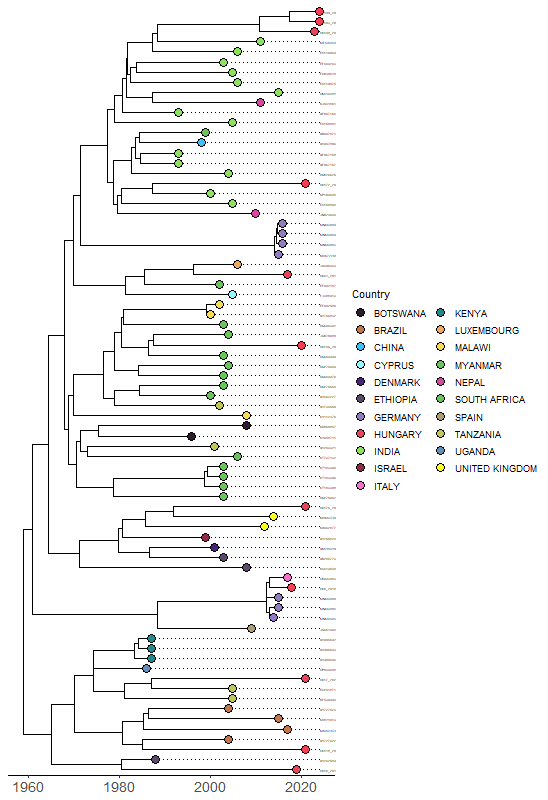
Supplementary Figure 18: Bayesian MCC tree from Hungarian and international HIV-1 sequences for subtype C. Tips are colored according to country of origin.


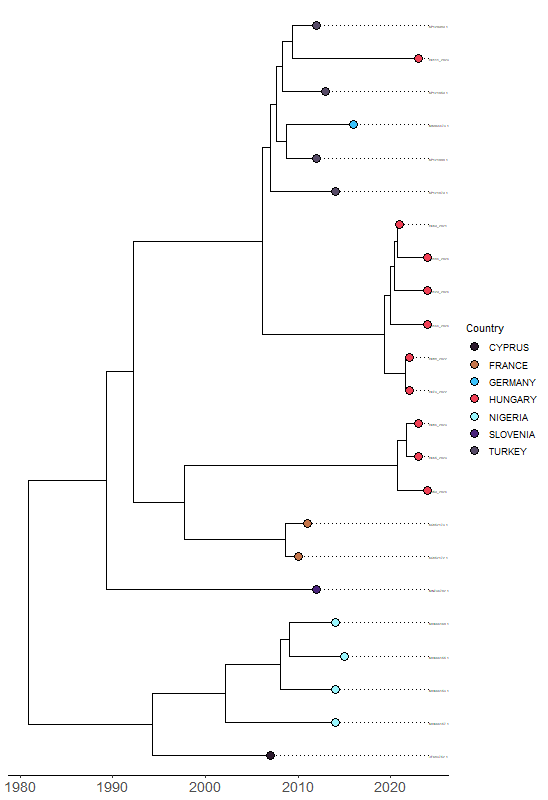
Supplementary Figure 19: Bayesian MCC tree from Hungarian and international HIV-1 sequences for CRF56_cpx. Tips are colored according to country of origin.


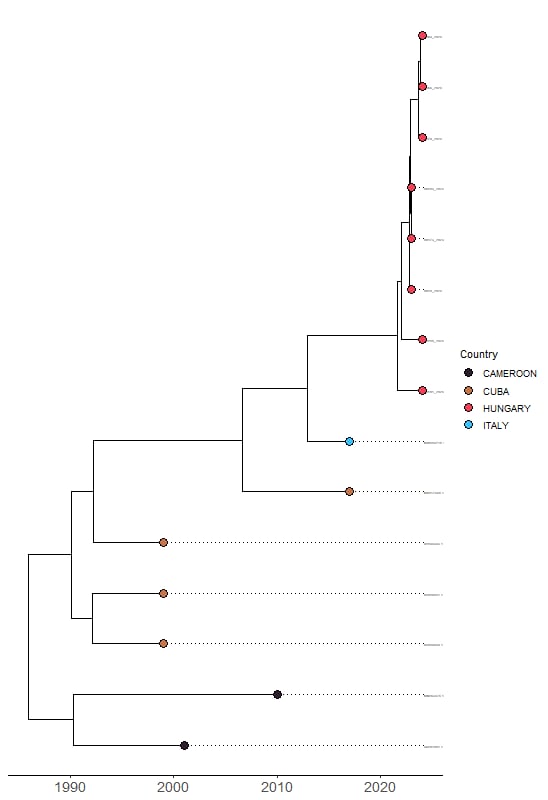
Supplementary Figure 20: Bayesian MCC tree from Hungarian and international HIV-1 sequences for CRF18_cpx. Tips are colored according to country of origin.


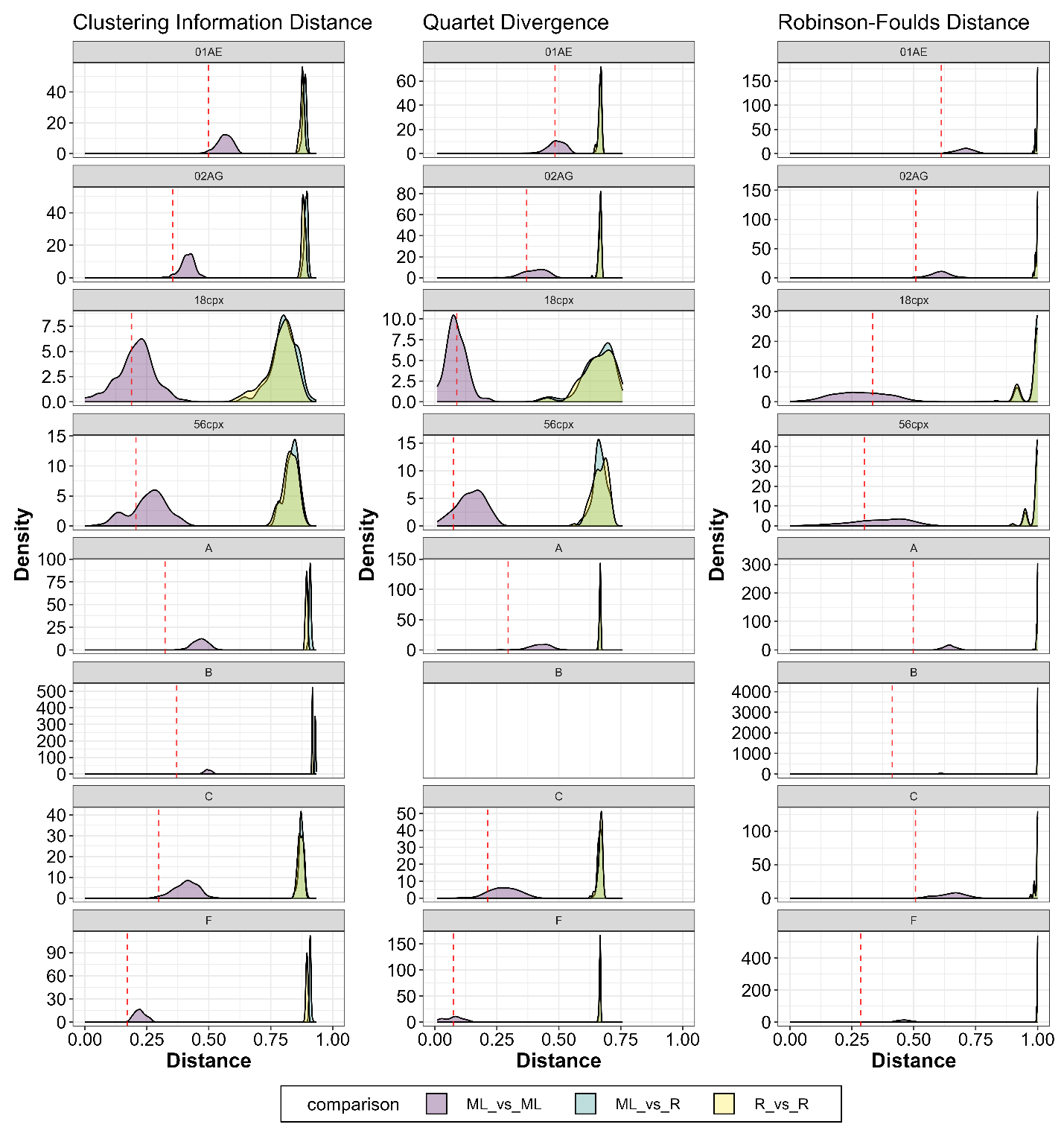
Supplementary Figure 21: Comparison of maximum likelihood (ML), Bayesian, and random phylogenetic trees by subtype constructed from Hungarian and international background sequences. Each panel shows subtype-specific density plots for four phylogenetic tree distance metrics: Robinson–Foulds Distance, Quartet Divergence, Kendall–Colijn Distance, and Mutual Clustering Information. Purple density curves represent distances between ML bootstrap trees; yellow curves represent distances between randomly generated trees with matched tip labels and sizes; green curves represent distances between ML bootstrap and random trees. A red dashed vertical line indicates the distance between the ML consensus tree and the Bayesian maximum clade credibility (MCC) tree. Abbreviations: ML – maximum likelihood; R – random**.**
